# Mapping the landscape of healthcare-associated infections in China, 2015–2019: a nation-wide observational study

**DOI:** 10.1016/j.lanwpc.2025.101775

**Published:** 2025-12-16

**Authors:** Hong-Wu Yao, Chen-Long Lv, Yao Tian, Yu-Zheng Zhang, Zheng-Hao Yu, Ming-Mei Du, Cheng-Xue Ma, Ji-Jiang Suo, Shuo Zhao, Yu Zhang, Yu-Bin Xing, An-Ran Zhang, Yan-Ling Bai, Bo-Wei Liu, Zhong-Qiang Yan, Ju-Yuan Liu, Meng Cai, Rui Huo, Jian Lin, Chun-Ping Chen, Bao-Gui Jiang, Qiang Xu, Jin-Jin Chen, Qing-Bin Lu, Yang Yang, Wei Liu, Li-Qun Fang, Yun-Xi Liu

**Affiliations:** a Department of Disease Prevention and Control, The First Medical Center, Chinese PLA General Hospital, Beijing, PR China; b State Key Laboratory of Pathogen and Biosecurity, Academy of Military Medical Science, Beijing, PR China; c National Center for Mental Health, China, Beijing, PR China; d National Institute of Hospital Administration, The China's National Health Commission, Beijing, PR China; e Department of Epidemiology, School of Public Health, Cheeloo College of Medicine, Shandong University, Jinan, PR China; f Hospital Infection Control Department, Beijing Hospital, National Center of Gerontology; Institute of Geriatric Medicine, Chinese Academy of Medical Sciences, Beijing, PR China; g HangZhou XingLin Information Technology CO., LTD, Hangzhou, PR China; h Department of Laboratorial Science and Technology, School of Public Health, Peking University, Beijing, PR China; i Department of Biostatistics, College of Public Health and Health Professions, and Emerging Pathogens Institute, University of Florida, Gainesville, FL, USA

**Keywords:** Healthcare-associated infection, Multi-drug resistant organisms, Risk factors, Spatiotemporal heterogeneity, Antimicrobial use

## Abstract

**Background:**

Healthcare-associated infections (HAIs), complicated by antimicrobial resistance continue to affect all countries with unprecedented threat, especially for developing countries. Our objective was to explore the epidemiological features, spatiotemporal heterogeneity and associated factors of HAIs and HAI related multi-drug resistant organisms (MDROs) in China.

**Methods:**

We used national surveillance data that were collected from 6867 sampled hospitals during 2015–2019 to determine the annual incidence of HAIs, key HAI-related quality indicators and associated factors.

**Findings:**

During 2015–2019, total of 4,959,230 HAIs were reported in sampled hospitals in China, with the overall incidence and prevalence estimated as 1.1 and 2.3 per 100 inpatients, which showed decline trend over time. Carbapenem-resistant *Acinetobacter baumannii* (CRAB) had the highest overall detection rate (51.1%), followed by methicillin-resistant *Staphylococcus aureus* (MRSA) (31.0%), carbapenem-resistant *Pseudomonas aeruginosa* (CRPA) (22.8%), and carbapenem-resistant *Klebsiella pneumonia* (CRKP) (12.4%). Detection rates of MRSA, carbapenem-resistant *Escherichia coli* (CREC), and vancomycin-resistant *Enterococcus faecium* (VREfm) reduced, in contrast with an increasing trend for CRKP. Higher risk of HAIs was associated with hospitals located in urban areas (incidence rate ratio [IRR]: 1.39, p < 0.001), in regions with higher GDP per capita (IRR: 1.03, p < 0.001), with more beds (IRR: 1.64 and 2.16 for 500−1500 and ≥ 1500 beds respectively, p < 0.001), as well as in south region of China (IRR: 1.23, p < 0.001). The reduced annual rates of HAIs were observed after 2017 (IRR: 0.92, p < 0.001), when 12 new standards targeted for HAIs were implemented.

**Interpretation:**

The study increases the understanding of HAIs and antimicrobial resistance. It highlights high-risk areas of HAIs and MDROs of concern, where targeted measures to continuously enhance management and policies implementation of HAIs are needed.

**Funding:**

The Foundation of State Key Laboratory of Pathogen and Biosecurity of China (Grant No. SKLPBS2443) and Infection Prevention and Control Research Project of “Gan·Dong China” (Grant No. GY2023022-A).

**Research in context**

**Evidence before this study**

We searched PubMed and Web of Science for peer-reviewed research publications and reports published in English and Chinese on the epidemiology of healthcare-associated infections (HAIs) in China. Our focus was on the epidemic characteristics, spatiotemporal heterogeneity, and associated factors of HAIs. The search terms were as follows: (*“healthcare-associated infection”* [MeSH Terms] OR *“healthcare associated infection”* [MeSH Terms] OR *“nosocomial infection”* [MeSH Terms] OR *“hospital-acquired infection”* [MeSH Terms] OR “*hospital acquired infection”* [MeSH Terms]) AND (*“China”* [MeSH Terms]) without any language restrictions. The publication date was limited to studies published from 2006 onward. Previous studies show that, despite a growing number of studies on the burden of HAIs in China, most have focused on point prevalence surveys (PPSs) conducted at individual hospitals or within specific regions. A limited number of multicenter studies have reported a comparable level of HAI burden across China between 2012 and 2018. However, these studies cannot be considered conclusively representative in China, and the spatiotemporal dynamics of the HAI burden across the entire nation in recent years, as well as the impact of administrative interventions on HAI rates, remain inadequately understood.

**Added value of this study**

In this study, we utilized national surveillance data collected from 6867 hospitals between 2015 and 2019 to examine the spatiotemporal dynamics of eight key HAI-related quality indicators, e.g., incidence rate of HAIs, detection rate of multi-drug resistant organisms (MDROs) of seven common antimicrobial-resistant bacteria associated with HAI, and others. We also analyzed the factors associated with HAIs in China. Furthermore, the study assessed the impact of policies designed to control HAIs on their incidence and patterns. Our findings revealed an overall HAI incidence of 1.1 per 100 inpatients, with a declining trend over time. The results also showed spatiotemporal variations and identified risk factors associated with HAIs caused by MDROs (e.g., CRAB, CRKP, and MRSA) and other HAI-related quality indicators. Notably, the study suggested that the implementation of well-developed healthcare policies and programs was associated with preventing and controlling HAIs, particularly following the implementation of 12 new specialized standards since 2017.

**Implications of all the available evidence**

By mapping the landscape of HAIs in China, this study provides a national overview of the burden and spatiotemporal heterogeneity of HAIs for the first time. Our findings elucidate critical knowledge gaps regarding the pattern of HAI burden and the detection rates of MDROs vary over time and across different regions of China. Evidence-based public health measures could prioritize on hospitals in higher-income areas with large bed capacities, particularly those in southern China, while simultaneously reinforcing surveillance and control strategies for MDROs with elevated detection rates. This study contributes to the understanding of HAIs and antimicrobial resistance, identifies high-risk areas for HAIs and MDROs, and underscores the necessity of targeted interventions as well as sustained improvements in management and policy implementation to effectively tackle the challenges posed by HAIs.

## Introduction

Healthcare-associated infections (HAIs), also known as nosocomial infections or hospital-acquired infections, affects hundreds of millions of patients every year worldwide.^1^ Although being evidence-based preventable with estimated to be preventable in up to 50.0% of cases or more. HAIs remain as one of the most frequent and severe concern in health care due to their frequent adverse consequences and heavy financial burdens.^1^, ^2^, ^3^, ^4^ Along with the increasing occurrence of emerging infectious diseases (e.g., COVID-19), acceleration of aging population and the emergence of new medical technology and so on,^5^, ^6^, ^7^, ^8^ the risk of HAI has been exacerbated and expanded globally. The heterogeneity as to the risk and effect of HAI was additionally observed, with developing regions afflicted with relatively higher burden and with on-rise trend due to its higher prevalence of HAI (10.1 per 100 patients) than that of developed regions (7.0 per 100 patients) according to recent data.^3^^,^^9^^,^^10^ While HAI surveillance systems are in place at national level in many developed countries, few developing countries reported a functioning national surveillance system, therefore, limited data are available from the vast majority of low- and middle-income countries.^3^^,^^11^

**Fig. 1:**
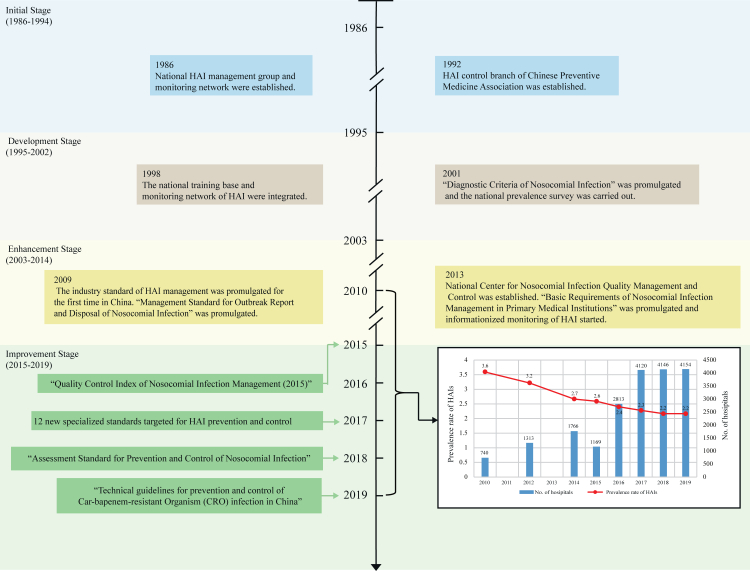
Timeline of major events for the prevention and control for HAI in China from 1986 to 2019. The prevalence rates and number of hospitals in 2010, 2012 and 2014 in the bar chart are from the literature, while those from 2015 to 2019 are calculated by this study.

As the largest developing country in the world, China has approximately 34,500 hospitals and over 200 million discharged patients annually, thus facing the prominent challenge and threat from HAIs with an estimated annual economic burden of US$1.5−US$2.3 billion.^12^, ^13^, ^14^ A literature review of studies published from January 2006 to April 2025 revealed that despite an increasing number of studies on HAI burden in China, most of them focused on point prevalence survey (PPS) at either a single hospital or a specific region, and few studies at national-level were published in English peer-reviewed journals.^13^, ^14^, ^15^, ^16^, ^17^, ^18^, ^19^, ^20^, ^21^ Two large multicentre studies calculated an estimation of the overall cross-sectional point prevalence of HAIs by 3.22% among 1313 hospitals in 2012 and 2.67% among 1766 hospitals in 2014 in China,^15^^,^^16^ and another two studies involving 52 hospitals (October 2014 to March 2015) and 189 hospitals (June 2017 to May 2018) reported prevalence of 3.7% and 1.24%, respectively.^17^^,^^18^ These multicentre studies revealed a comparable level of HAI burden in China during 2012 and 2018. The China’s National Health Commission (NHC) has reinforced legislation and taken some critical measures to control HAI from 1986, mainly including regulatory managements, coordinated interventions, comprehensive and targeted surveillance, implementation of infection control bundles, antimicrobial stewardship programs and so on (Fig. 1, Supplementary Table S1). Whether the level of HAIs was affected by the administration of these projects remains elusive.^13^^,^^19^

Most hospitals in China have progressively established electronic surveillance systems since the early 2000s, and National Nosocomial Infection Management and Quality Control Center (NNIMQCC) with a four-hierarchy (national-provincial-municipal-institutional) surveillance system had been operating by NHC of China since 2013.^19^ The surveillance of HAIs based on minimum data set and quality indicators (QIs) was advocated and developed rapidly in thousands of hospitals of China.^19^ The NNIMQCC issued a targeted surveillance notice, focusing on collecting key QIs of HAIs from each hospital enrolled in the surveillance system every year since 2015,^22^ which makes it possible to describe a complete picture of HAI burden and antimicrobial resistance among the nosocomial isolates in China. In the current study, we aim to explore the epidemiological features, spatiotemporal heterogeneity and associated factors of HAIs and HAI related multi-drug resistant organisms (MDROs) in China during 2015–2019 by analyzing the surveillance data from NNIMQCC. We further assessed the effect of policies to control the HAIs on affecting the magnitude and pattern of HAIs. By analyzing the surveillance data in China, it will provide reference for relevant prevention and control measures.

## Methods

### Surveillance data processing

Since 2015, when the NNIMQCC initiated the collection of surveillance data on HAIs, which encompassed all patients including both adults and children, all participating hospitals were required to submit annual statistic data on the 13 HAI related QIs, which were either extracted directly or calculated based on their HAI surveillance systems. Among these, eight key QIs with available data were included in this study. These QIs encompassed incidence rate of HAIs, detection rate of MDROs including seven common antimicrobial-resistant bacteria associated with HAI, incidence rate of surgical site infections (SSI) in patients with class I/Clean wounds, incidence density of central line-associated bloodstream infection (CLABSI), incidence density of catheter-associated urinary tract infection (CAUTI), incidence density of ventilator-associated pneumonia (VAP), inpatient antimicrobial usage rate, and the bacterial culture rate before therapeutic use of antimicrobial agents, which was emphasized in one of 12 new standards (implemented in 2017) named regulation for prevention and control of healthcare associated infection in intensive care unit (Supplementary Table S1). The MDROs which may not be the strictly multidrug-resistant pathogens, mainly include carbapenem-resistant *Acinetobacter baumannii* (CRAB), carbapenem-resistant *Escherichia coli* (CREC), carbapenem-resistant *Klebsiella pneumoniae* (CRKP), carbapenem-resistant *Pseudomonas aeruginosa* (CRPA), methicillin-resistant *Staphylococcus aureus* (MRSA), vancomycin-resistant *Enterococcus faecalis* (VREfs) and vancomycin-resistant *Enterococcus faecium* (VREfm). The definition and calculation rule of each of the eight key HAI related QIs were detailed in Supplementary Table S2. HAI was defined as an infection occurring in a patient during the process of care in a health-care facility which was not present or incubating at the time of admission according to the Chinese Nosocomial Infection Diagnosis Criterion (2001) (detailed in Supplementary Table S3) published by the National Health Commission of the People’s Republic of China.^23^ This includes infections acquired in the hospital (after 48 h’ admission), as well as occupational infections among staff members. As shown in Supplementary Table S3, the Chinese Diagnosis Criterion prioritizes clinical signs (e.g., localized inflammation, purulent drainage) and physician judgment over mandatory etiological confirmation. Most infections can be diagnosed in the absence of positive microbiological cultures, with the phrase “WITH or WITHOUT a positive microbiological culture” frequently referenced across various infection types (e.g., superficial or deep surgical site infections). In contrast, US standards typically require more rigorous laboratory confirmation (e.g., quantitative cultures, paired sera), higher pathogen thresholds, and objective evidence to support clinical signs. For each region, the QIs were computed using the aggregated data for both numerators and denominators. The occupational infections among staff were not included in this study.

The surveillance data of HAIs and the baseline information of participating hospitals were collected from 6867 hospitals distributed in all 31 provinces (autonomous regions and municipalities) of the mainland of China during 2015–2019. Hong Kong, Macau, and Taiwan were not included due to the lack of data. Data were sorted and cleaned following standard criteria and procedures, and a flowchart and the details of data sorting and cleaning procedures for calculating each QI separately were provided in the Supplementary Figure S1. Each hospital was geo-referenced and linked to a digital map of China (http://www.geodata.cn) using ArcGIS 10.7 software (ESRI Inc., Redlands, CA, USA). The data of carbapenem antimicrobial consumption from 2015 to 2019 were collected from Chinese Pharmaceutical Association (http://www.cpa.org.cn).

### Statistical analysis

#### The estimation of prevalence rate of HAI

The incidence rate of HAI was calculated as the number of patients acquiring a new infection per 100 inpatients. There is no relevant data on the prevalence of HAIs in this study. In order to facilitate meaningful comparisons across different studies, and considering the importance and variation of raw HAI prevalence during the surveillance period, we estimated the rough prevalence rate of HAI from the incidence rate using the following formula:^24^

Prevalence rate = Incidence rate $\times \frac{\mathrm{LN}\;-\;\mathrm{INT}}{\text{LA}}$

where LA is the average length of hospital stay of all inpatients, LN is the median length of hospital stay of inpatients who acquire one or more HAIs and INT is the median interval between hospital admission and onset of the first HAI. From 2015 to 2019, the values of LA were 9.6, 9.4, 9.3, 9.3 and 9.1, respectively, as extracted from the National Bureau of Statistics (https://www.stats.gov.cn/). The values of LN and INT were extracted from the published literature and respectively identified as 30.4 and 10 in this study.^17^^,^^25^^,^^26^

#### The trend analyses of key quality indicators of HAI

The annual rate of each of QIs was calculated as described in Supplementary Table S2, based on which the temporal trend from 2015 to 2019 was tested by linear regression. We also conducted a sub-analysis by exclusively using data from the 476 hospitals that participated throughout all study years to explain the inclusion and selection bias. Inter-group difference in the HAI-QIs was compared by Chi-square test. Thematic maps were created to display the spatiotemporal dynamics of HAI-QIs by using ArcGIS 10.7 software (ESRI Inc., Redlands, CA, USA). The annual change of device-associated infection rates between different provinces was showed by using heat map. If not otherwise specified, all tests were considered as significant at p value < 0.05. Data were analyzed by SAS (version 9.4, SAS INSTITUTE INC) and R software (version 4.0.3, R Development Core Team 2020).

#### The risk factor analyses of key quality indicators of HAI

The analysis on risk factors of HAI incidence rate and MDRO detection rate was conducted at the hospital level using the median rates to represent the average level of corresponding indicators in hospitals of different subgroups. We used a generalized estimation equation (GEE) model to assess the impacts of hospital level, number of beds, region of hospital (North/South, Supplementary Figure S2), urban/rural area and developed/developing regions (with GDP per capita ≥US$12,535/<US$12,535 based on the World Bank’s classification standard of GDP per capita in 2020) where hospital was distributed, 12 new standards targeted for HAI (before/after, indicates 2015–2017 and 2018–2019 respectively), annual number of inpatients and provincial GDP per capita on the annual incidence rate of HAI, the detection rates of MDROs and incidence density of three device-associated infections. These variables were chosen mainly considering the potential impacts of macro factors such as hospital characteristics, geographical location, economic regions and national policies. Selection of optimal model was based on the Quasi-likelihood under Independence Model Criterion (QIC) combined with p value.^27^

### Ethics approval

This study was ethically approved by the Hospital Ethical Committee of Chinese PLA General Hospital (Approval No.: S2019-142-02).

### Role of the funding source

This study is funded by the Foundation of State Key Laboratory of Pathogen and Biosecurity of China (Grant No. SKLPBS2443) and Infection Prevention and Control Research Project of “Gan·Dong China” (Grant No. GY2023022-A). The findings and conclusions in this report are those of the authors and do not reflect the views of the China's National Health Commission or the National Nosocomial Infection Management and Quality Control Center. The funders had no role in the study design, decision to publish or preparation of the manuscript.

## Results

### The overall incidence and prevalence of HAIs

**Fig. 2:**
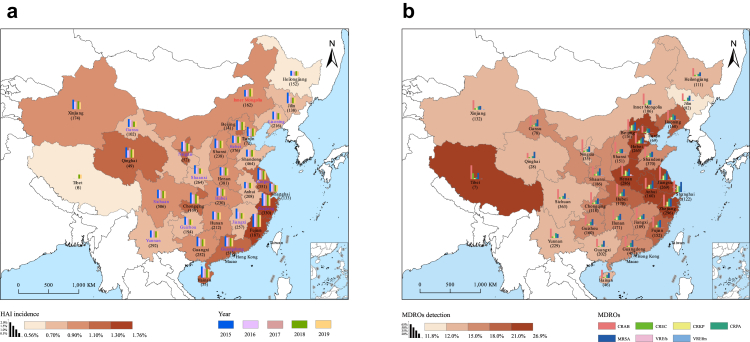
HAI incidence and overall MDROs detection mapped in China, 2015–2019. (a) The bar charts indicate the annual incidence rate of HAI, with sum level marked by the background color both at the province level. The provinces with name in red color had a significant increase of HAI incidence from 2015 to 2019, name in blue had a significant decrease, and name in black had no significant change; (b) The bar charts indicate the average detection rate of seven MDROs during 2015–2019 at the province level, with the overall detection rate of MDROs marked by the background color. The total number of reporting hospitals in each province during 2015−19 is noted in brackets. HAI: healthcare-associated infection; MDRO: multi-drug resistant organism; CRAB: carbapenem-resistant *A. baumannii*; CREC: carbapenem-resistant *E. coli*; CRKP: carbapenem-resistant *K. pneumoniae*; CRPA: carbapenem-resistant *P. aeruginosa*; MRSA: methicillin-resistant *S. aureus*; VREfs: vancomycin-resistant *E. faecalis*; VREfm: vancomycin-resistant *E. faecium*.

During 2015–2019, a total of 4,959,230 HAIs were reported in a pooled 6867 hospitals located in all 31 provinces in the mainland of China. The overall incidence is 1.1 per 100 inpatients during the study period, and the overall prevalence was roughly calculated to be 2.3 per 100 inpatients (Table 1). The annual incidence rates declined by 16.7% from 1.2% in 2015 to 1.0% in 2019 and the annual prevalence rates decreased from 2.6% in 2015 to 2.2% in 2019 (both p values for linear trends <0.05). The median of annualized incidence rates was 1.0% [interquartile range (IQR): 0.9%–1.2%] for the 31 provinces during 2015–2019, which ranged between 0.6% and 1.8% with overall prevalence ranged between 1.3% and 3.9% (Fig. 2a).

**Table 1:** The annual incidence of HAI quality indicators in China, during 2015–2019.

	Total	2015	2016	2017	2018	2019	β coefficient of linear trend (95% CI)	p value^a^
Occurrence of HAI								
Number of eligible hospitals	6867	1169	2813	4120	4146	4154		
Number of inpatients	459,187,963	39,088,211	81,817,000	111,287,619	116,960,686	124,177,449		
Number of new HAIs	4,959,230	480,785	899,987	1,168,520	1,192,999	1,216,939		
Incidence rate of HAIs (%)	1.1	1.2	1.1	1.1	1.0	1.0	−0.96 (−1.49, −0.43)	0.011↓
Non-tertiary (number of eligible hospitals)	0.7 (4474)	0.7 (616)	0.7 (1818)	0.7 (2527)	0.7 (2444)	0.6 (2398)	−1.00 (−1.15, −0.84)	<0.001↓
Tertiary (number of eligible hospitals)	1.3 (2393)	1.5 (553)	1.4 (995)	1.3 (1593)	1.2 (1702)	1.2 (1756)	−0.99 (−1.21, −0.78)	<0.001↓
Average length of hospital stay (days)	9.3	9.6	9.4	9.3	9.3	9.1		
Prevalence calculated from Incidence (%)	2.3	2.6	2.4	2.3	2.2	2.2	−0.95 (−1.52, −0.39)	0.013↓
Detection of CRAB								
Number of eligible hospitals	3963	864	1615	2375	2497	2595		
Number of *Acinetobacter baumannii* isolates	2,046,609	219,562	355,353	452,986	497,512	528,734		
Number of CRAB isolates	1,045,817	114,897	183,078	228,033	251,741	268,068		
Detection rate of CRAB (%)	51.1	52.3	51.5	50.3	50.6	50.7	−0.81 (−1.89, 0.28)	0.099
Detection of CREC								
Number of eligible hospitals	4160	747	1452	2251	2395	2528		
Number of *Escherichia coli* isolates	3,857,235	373,780	646,655	919,316	1,048,239	1,159,578		
Number of CREC isolates	203,662	26,501	36,924	47,069	46,437	46,731		
Detection rate of CREC (%)	5.3	7.1	5.7	5.1	4.4	4.0	−0.97 (−1.39, −0.56)	0.005↓
Detection of CRKP								
Number of eligible hospitals	4765	1001	1872	2886	3061	3151		
Number of *Klebsiella pneumoniae* isolates	3,793,475	330,133	535,064	804,131	936,041	1,035,816		
Number of CRKP isolates	471,529	34,697	58,643	103,170	127,208	147,811		
Detection rate of CRKP (%)	12.4	10.5	11.0	12.8	13.6	14.3	0.98 (0.61, 1.35)	0.004↑
Detection of CRPA								
Number of eligible hospitals	4457	961	1807	2692	2862	2980		
Number of *Pseudomonas aeruginosa* isolates	2,806,277	266,928	456,229	619,478	717,454	737,131		
Number of CRPA isolates	640,673	60,833	99,686	144,772	167,095	168,287		
Detection rate of CRPA (%)	22.8	22.8	21.9	23.4	23.3	22.8	0.40 (−1.29, 2.08)	0.510
Detection of MRSA								
Number of eligible hospitals	4774	1002	1928	2887	3087	3148		
Number of *Staphylococcus aureus* isolates	2,198,750	199,444	344,154	510,310	574,196	611,903		
Number of MRSA isolates	682,492	67,831	109,441	154,981	168,871	181,368		
Detection rate of MRSA (%)	31.0	34.0	31.8	30.4	29.4	29.6	−0.93 (−1.62, −0.23)	0.024↓
Detection of VREfs								
Number of eligible hospitals	2920	642	1093	1608	1796	1842		
Number of *Enterococcus faecalis* isolates	550,484	52,748	87,818	121,182	140,089	146,258		
Number of VREfs isolates	6826	691	966	1333	1569	2267		
Detection rate of VREfs (%)	1.2	1.3	1.1	1.1	1.1	1.6	0.40 (−1.29, 2.08)	0.505
Detection of VREfm								
Number of eligible hospitals	2773	604	1042	1528	1682	1766		
Number of *Enterococcus faecium* isolates	525,135	52,556	84,385	124,203	148,818	164,028		
Number of VREfm isolates	11,658	1645	2059	2571	3021	2362		
Detection rate of VREfm (%)	2.2	3.1	2.4	2.1	2.0	1.4	−0.97 (−1.45, −0.48)	0.008↓
New occurrence of SSI in patients with class I/Clean wounds								
Number of eligible hospitals	4985	1027	2051	2939	3016	2989		
Number of surgery with class I/Clean wounds	41,730,000	3,870,313	6,990,938	9,280,303	10,407,419	11,328,214		
Number of SSI in patients with class I/Clean wounds	129,363	12,385	22,371	30,625	32,263	31,719		
Incidence rate (%)	0.3	0.3	0.3	0.3	0.3	0.3	−0.77 (−1.95, 0.41)	0.131
New occurrence of CLABSI								
Number of eligible hospitals	4861	1146	2118	3056	3237	3332		
Total days of inpatients using central intravascular catheter	84,786,076	6,990,385	11,823,810	19,918,571	24,003,077	28,102,778		
Number of CLABSI	66,981	7270	9932	13,943	15,602	20,234		
Incidence density (events/1000 days)	0.8	1.0	0.8	0.7	0.7	0.7	−0.83 (−1.85, 0.19)	0.081
New occurrence of CAUTI								
Number of eligible hospitals	6485	1328	2593	3943	4132	4253		
Total days of inpatients using catheter	124,829,560	11,032,768	17,861,582	27,987,037	33,375,839	39,596,212		
Number of CAUTI	198,479	19,528	31,615	45,339	49,730	52,267		
Incidence density (events/1000 days)	1.6	1.8	1.8	1.6	1.5	1.3	−0.97 (−1.44, −0.49)	0.007↓
New occurrence of VAP								
Number of eligible hospitals	4521	1103	2017	2935	3063	3183		
Total days of inpatients using ventilator	25,509,102	2,637,188	4,203,085	6,093,068	6,808,412	7,665,232		
Number of VAP	210,195	28,508	39,509	47,465	50,178	44,535		
Incidence density (events/1000 days)	8.2	10.8	9.4	7.8	7.4	5.8	−0.99 (−1.27, −0.71)	0.002↓
Inpatient’s antimicrobial usage								
Number of eligible hospitals	8652	1301	3021	5195	5283	5285		
Number of inpatients	482,637,839	40,672,643	77,312,508	111,022,427	120,845,735	130,656,895		
Number of inpatients with antimicrobial usage	219,503,689	17,684,465	35,950,316	51,836,371	55,419,854	58,612,683		
Inpatient’s antimicrobial usage rate (%)	45.5	43.5	46.5	46.7	45.9	44.9	0.25 (−1.53, 2.03)	0.683
Bacterial culture before therapeutic use of antimicrobial agents								
Number of eligible hospitals	5921	947	2255	2985	3036	3211		
Number of inpatients	121,995,279	10,125,147	22,751,265	27,554,869	30,234,831	33,349,110		
Number of patients for bacterial culture	50,469,447	4,579,604	9,569,182	11,148,700	11,752,279	13,419,682		
Bacterial culture rate (%)	41.4	45.2	42.1	40.5	38.9	40.2	−0.86 (−1.81, 0.10)	0.065

The definition and proportion for each HAI quality indicators are provided in Supplementary Table S2.

HAI, healthcare-associated infection; CRAB, carbapenem-resistant *A. baumannii*; CREC, carbapenem-resistant *E. coli*; CRKP, carbapenem-resistant *Klebsiella pneumonia*; CRPA, carbapenem-resistant *P. aeruginosa*; MRSA, methicillin-resistant *S. aureus*; VREfs, vancomycin-resistant *E. faecalis*; VREfm, vancomycin-resistant *E. faecium*; SSI, surgical site infections; CLABSI, central line-associated bloodstream infection; CAUTI, catheter-associated urinary tract infection; VAP, ventilator-associated pneumonia.

↑: Significant increasing tendency. ↓: Significant decreasing tendency.

a Linear regression for trends.

Among the seven main MDROs (Table 1), CRAB had the highest overall detection (51.1%), followed by MRSA (31.0%), CRPA (22.8%), and CRKP (12.4%), others had a low detection including CREC (5.3%), VREfm (2.2%) and VREfs (1.2%). The temporal trends from 2015 to 2019 varied, with three of them (MRSA, CREC, and VREfm) showing a significant reduction of detection rates from 2015 to 2019 by 13.0%, 43.0%, and 54.0%, respectively (all p < 0.05 for linear regression for trends), in contrast, a significant increase from 10.5% in 2015 to 14.3% in 2019 was shown for CRKP (p < 0.01 for linear regression for trends), and no significant change was found for other three MDROs, except for a rebound for VREfs in 2019.

In terms of the infection type of HAI, the incidence densities of VAP and CAUTI had persistently reduced from 10.8 infections/1000 ventilator-days and 1.8 infections/1000 urinary catheter-days in 2015 to 5.8 infections/1000 ventilator-days and 1.3 infections/1000 urinary catheter-days in 2019, respectively (both p < 0.01). In contrast, CLABSI and SSI in patients with class I/Clean wounds were shown not to have a linear decrease pattern with average annualized incidence of 0.8/1000 catheter-days and 0.3/100 patients. The rate of antimicrobial usage among inpatients stabilized at about 45.0% every year, and the bacterial culture rate before therapeutic use of antimicrobial agents, of which average value was 41.4%, dropped persistently from 45.2% in 2015 to 38.9% in 2018, but a rebound was seen in 2019 (40.2%) (Table 1). To demonstrate the robustness of our findings, we validated the trend test results using the Mann-Kendall method, and the results indicated that, apart from the annual trend in detection rate of MRSA—which shifted from a significant decline to non-insignificance—most other results were consistent with those derived from linear regression analysis (Supplementary Table S4).

The sub-analysis, conducted exclusively with data from the 476 hospitals that participated throughout all study years, also demonstrated a slightly decreasing trend in the annual incidence rate and prevalence of HAIs (from 1.4% in 2015 to 1.2% in 2019; Supplementary Table S5). This finding is consistent with the results of the primary analysis (Table 1). Among the other QIs, detection rates of four MDROs (CREC, CRKP, VREfs, and VREfm) as well as four QIs (incidence rate of SSI in patients with class I/Clean wounds, incidence density of CAUTI, incidence density of VAP, and inpatient antimicrobial usage rate) showed trends that were consistent with the primary analysis. In addition, detection rates of two MDROs (CRAB and CRPA) shifted from non-significant trends in the primary analysis to significantly increasing trends. Two other QIs (incidence density of CLABSI and bacterial culture rate before therapeutic use of antimicrobial agents) transitioned from non-significant trends in the primary analysis to significantly decreasing trends. Conversely, detection rates of MRSA exhibited a change from a significantly decreasing trend in the primary analysis to a non-significant trend.

### Spatiotemporal heterogeneity of HAIs

Both incidence density and dynamic pattern of HAIs showed heterogeneity among provinces. Higher annualized average incidence rates of HAIs were observed in the southeast coastal provinces e.g., Zhejiang and Fujian provinces, than those in the inland provinces. Beijing, Chongqing, Qinghai, and Ningxia provinces were few exceptions among inland provinces presenting with high incidence rates of HAIs (>1.1%) (Fig. 2a). Among all the 31 studied provinces, 17 provinces showed non-significant change, 13 had a significant decreasing pattern of HAIs from 2015 to 2019, while only Inner Mongolia displayed an increasing pattern (Fig. 2a).

**Fig. 3:**
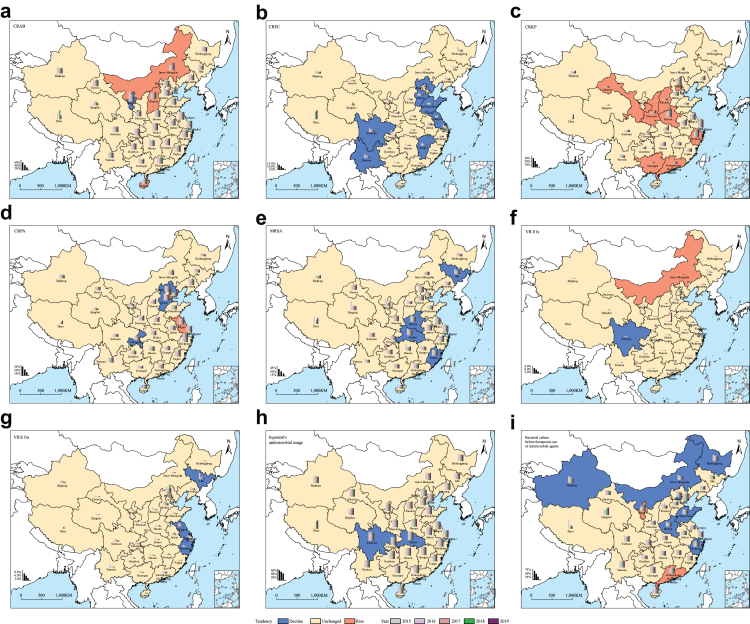
Spatiotemporal pattern of detection rates for seven species of MDROs, bacterial culture rate and inpatient antimicrobial usage rate in China, 2015−2019. (a) Carbapenem-resistant *Acinetobacter baumannii* (CRAB); (b) carbapenem-resistant *Escherichia coli* (CREC); (c) carbapenem-resistant *Klebsiella pneumoniae* (CRKP); (d) carbapenem-resistant *Pseudomonas aeruginosa* (CRPA); (e) methicillin-resistant *Staphylococcus aureus* (MRSA); (f) vancomycin-resistant *Enterococcus faecalis* (VREfs); (g) vancomycin-resistant *Enterococcus faecium* (VREfm); (h) inpatient antimicrobial usage; (i) bacterial culture rate before therapeutic use of antimicrobial agents. The background with red (blue) color in provinces indicates a significant linear increasing (decreasing) trend of annual detection rate for specific species of MDROs. The bar charts indicate the annual detection rates of specific species of MDROs in each province.

When seven types of MDROs were estimated as a whole, provinces in eastern China (especially in Yangtze River Delta) had high rates of antimicrobial resistance (>21.0%) compared with those in western and northeastern China. Henan and Beijing had shown relatively higher rates than other provinces in western and northeastern China. The higher detection rate of MDROs in eastern China was mostly attributed to the higher detection rates of CRKP and CRPA, rather than other MDROs (Fig. 2b). Almost all provinces had a highest detection rate of CRAB among the seven MDROs, with the medians of 51.0% (range: 24.8%–65.7%), followed by MRSA with the median of 29.1% (range: 17.8%–48.1%). However, Qinghai Province in northwestern China was the only province with a higher detection rate of MRSA than that of CRAB (Fig. 2b and 3).

For each type of MDROs, a high spatial heterogeneity was observed for their dynamic changes (Fig. 3a–g). In most provinces, no significant annual change in their detection rates was observed (in yellow background in each panel of Fig. 3). For the remaining provinces, a consistent decrease in the detection rate was observed for MRSA, CREC and VREfm (in blue background in Fig. 3b–e and g), and a generally observed increase in the detection rate was observed for CRKP (in red background in Fig. 3c), while heterogeneity was observed for other MDROs, i.e., CRAB, CRPA and VREfs (Fig. 3a–d and f). Notably, in Inner Mongolia located in northern China, both CRAB and VREfs were significantly increased for their detection (Fig. 3a and f), along with a significant decrease of bacterial culture rate before therapeutic use of antimicrobial agents (Fig. 3i). Other provinces in northern China also displayed significant decrease of bacterial culture rate from 2015 to 2019 (Fig. 3i). The rate of inpatient antimicrobial usage was kept at stable level in most provinces of China, with exception in Sichuan and Hubei provinces where a linear drop was observed (Fig. 3h).

For the three device-associated infections, a significant incidence density reduction of CLABSI, CAUTI, and VAP were identified in 10, 11 and 15 provinces, respectively (Supplementary Figure S3), with only one province decreasing in all three device-associated infections while 11 decreasing in two and no change in the third, and the others had displayed comparable infection density across the years, with the only exception of a significantly increasing trend for CAUTI only in Henan Province. Among the three device-associated infections, CAUTI and VAP were shown with significant decrease after 2017 (adjusted incidence rate ratio [IRR]: 0.86 and 0.61, p = 0.017 and p < 0.001) (Supplementary Table S6).

### The diverse pattern of HAI quality indicators stratified by subgroups

**Fig. 4:**
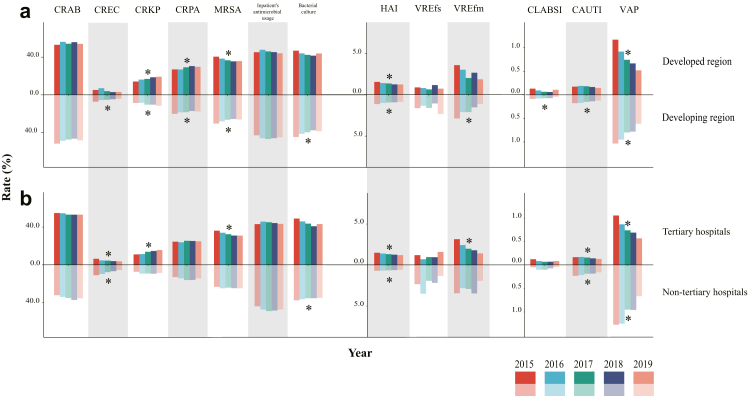
Comparison of annual trend of HAI related quality indicators (QIs) between different subgroups. (a) Developed region vs. developing region. (b) Tertiary hospitals vs. non-tertiary hospitals. ∗indicates a significant linear trend over the years during 2015–2019.

When annual QIs were measured within subgroups, a high heterogeneity of the level and dynamic trend was observed regarding hospital level and local income level. Notably, the developed region seemed to have a lower detection rate of CREC and VREfs, but with a higher rate for MRSA, CRPA, CRKP and VREfm by compared to the developing region (Fig. 4a). In addition, tertiary hospitals were shown to have higher detection rates of CRAB, MRSA, CRPA and CRKP, while the non-tertiary hospitals had higher detection rates of CREC, VREfm, VREfs and higher incidence density of VAP and CAUTI (Fig. 4b), which might be partially attributed to the differences in infrastructure, medical equipment, professionals, adherence to national standards, and compliance with local guidelines for HAI across hospitals of varying levels.

When the incidence rates from 2015 to 2019 were observed, only two QIs (incidence rates of HAI and VAP) showed significant decreases that were observed consistently in most subgroups in terms of hospital level and local income level (Fig. 4). The linearly decrease pattern of CREC and CAUTI was significant merely in the developing region. There was significant increase for the detection rate of CRKP, but only limited in tertiary hospitals, rather than non-tertiary hospitals. An inverse subgroup trend over time was observed for CRPA in that a significant increase was observed in developed region, while a decrease was shown in developing region (Fig. 4a). Although with comparable antimicrobial use between subgroups of hospital level and local income level, a significant decrease of bacterial culture rate was observed in local developing region and in non-tertiary hospitals than their counterpart groups (Fig. 4a and b). We also conducted adjustment for multiple outcomes using the Benjamini-Hochberg method. By compared with the results before adjustment, we observed that the annual trends of some QIs were no longer significant, including detection rates of CRPA and MRSA in both developed and developing region, bacterial culture rate and incidence density of CLABSI in the developing region, detection rate of CREC in tertiary hospitals, and bacterial culture rate in non-tertiary hospitals (Supplementary Figure S4).

### Risk factors of HAI quality indicators

All the seven variables included in the univariate GEE model were significantly associated with the incidence rate of HAI (Table 2). Among them the number of inpatients was not significant in the multivariate analysis and excluded. Hospitals with more beds and better resources (located in South China, urban, developed region) were related to higher incidence rates with the adjusted IRRs estimated as 1.23 to 2.16. Number of beds and number of inpatients were significantly related to the detection of almost all species of MDROs (Supplementary Table S7-S13). When each species of MDRO was separately analyzed, significant lower rate of CREC (adjusted IRR: 0.70, p < 0.001), higher rates of CRPA (adjusted IRR: 1.59, p = 0.008) and MRSA (adjusted IRR: 1.36, p = 0.003) were observed in urban than in rural areas after adjustment for other covariates. In addition, the quadratic GDP per capita were significantly associated with the detection rates of CRAB, CRKP, CRPA, MRSA and VREfm after multivariate adjustment, indicating the detection rates of three MDROs are nonlinearly associated with the increase of GDP per capita. Based on the multivariate analysis, number of beds, region of hospital and number of inpatients were related to the increased incidence of all three device-associated infections, with the adjusted IRR ranging from 1.44 to 2.88 (Supplementary Table S14-S16).

**Table 2:** The risk factors of incidence rate of healthcare-associated infection (HAI) in China.

	No. of hospitals	Incidence rate, Median (IQR, %)	Univariate analysis		Multivariate analysis	
			Crude IRR (95% CI) (95% CI)	p value	Adjusted IRR (95% CI)	p value
Hospital level						
Non-tertiary	4592	0.5 (0.2, 0.9)	1	–		
Tertiary	2392	1.0 (0.6, 1.6)	1.94 (1.86, 2.03)	<0.001		
Number of beds						
<500	4580	0.5 (0.2, 0.9)	1	–	1	–
500−1500	2062	1.0 (0.6, 1.5)	2.01 (1.92, 2.10)	<0.001	1.64 (1.55, 1.73)	<0.001
≥1500	248	1.2 (0.9, 1.9)	2.84 (2.61, 3.09)	<0.001	2.16 (1.97, 2.36)	<0.001
Region of hospital						
North	2981	0.6 (0.3, 1.1)	1	–	1	–
South	3885	0.8 (0.4, 1.3)	1.26 (1.20, 1.32)	<0.001	1.23 (1.18, 1.29)	<0.001
Urban area where hospital was distributed						
No	3395	0.5 (0.2, 0.9)	1	–	1	–
Yes	3471	0.9 (0.5, 1.5)	1.87 (1.79, 1.96)	<0.001	1.39 (1.31, 1.46)	<0.001
12 new standards targeted for HAI						
Before (2015–2017)	5177	0.7 (0.4, 1.3)	1	–	1	–
After (2018–2019)	5491	0.7 (0.3, 1.1)	0.94 (0.92, 0.96)	<0.001	0.92 (0.90, 0.94)	<0.001
Number of inpatients (10 thousand people), Continuous			1.03 (1.02, 1.05)	<0.001	No significant and excluded	
<2	4284	0.5 (0.2, 1.0)				
2−2.99	1417	0.7 (0.4, 1.1)				
3−3.99	988	0.8 (0.5, 1.2)				
≥4	1321	1.0 (0.7, 1.6)				
Provincial GDP per capita (10 thousand yuan), Continuous			1.04 (1.03, 1.04)	<0.001	1.03 (1.02, 1.03)	<0.001
<4	1999	0.6 (0.3, 1.0)				
4−5.99	2781	0.6 (0.3, 1.0)				
6−7.99	1635	0.7 (0.4, 1.3)				
≥8	2310	0.9 (0.5, 1.5)				

GDP: gross domestic product; IRR: incidence rate ratio.

Hospital level was excluded due to its high correlation with the area (urban/rural) where hospital was distributed.

The annual rates of HAI indicators were also evaluated for the change after the implementation of 12 new specialized standards for HAI in 2017. After adjustment for the covariates, the decline for the median incidence rate of HAI from 0.74 to 0.65 were determined as significant (adjusted IRR: 0.88, p < 0.001) (Supplementary Table S6). The increase in the detection rates after 2017 were determined as significant for CRAB (adjusted IRR: 1.20, p = 0.035), CRKP (adjusted IRR: 1.27, p = 0.015), CRPA (adjusted IRR: 1.29, p = 0.006), while the decreases were significant for CREC (adjusted IRR: 0.89, p = 0.014), VREfs (adjusted IRR: 0.70, p = 0.005), VREfm (adjusted IRR: 0.52, p < 0.001).

## Discussion

To our knowledge, the current study represents one of the first attempts to describe a continuous epidemiological picture and map the spatiotemporal heterogeneity of HAIs based on nation-wide surveillance data from a large number of sampled hospitals, especially when compared to previous PPSs in China.^15^^,^^16^ The effectiveness of healthcare policies and programs conducted in China has also been roughly assessed, which helps further prioritize strategies for HAI prevention.

Compared to the international study of HAIs, the incidence (1.1%) and prevalence (2.3%) of HAIs in China were low. In Asia, nation-wide surveillance is less common and the overall estimated prevalence rates of HAIs were tend to be higher, such as Southeast Asian countries (9.0% during 2000–2012), Saudi Arabia (6.8% in 2017), Japan (7.7% in 2016), and Vietnam (7.8% in 2008).^3^^,^^9^^,^^28^, ^29^, ^30^ The overall estimated prevalence rates were also higher in European countries (6.0% during 2011–2012 and 6.5% during 2016–2017).^31^ The low incidence and prevalence of HAI in China have been reported and explained in some studies, which could be partly impacted by the divergent definitions of HAI between China, US CDC and ECDC (Supplementary Table S3), particularly for lower respiratory tract infection.^32^^,^^33^ The broader definition of respiratory tract infections in China, combined with the extended use of perioperative antibiotics, is likely to result in a higher prevalence of respiratory tract infections and a lower prevalence of SSIs.^34^ Additionally, the differences may be explained by the large denominator (long length of stay) in China, which results from expanded insurance coverage for hospital services, leading patients to prefer receiving in-hospital treatment.^35^ Furthermore, the high antimicrobial use, combined with an overreliance on microbiology for case definition, the lower bacterial culture rate before the use of therapeutic antibiotics, low knowledge about infection prevention and control among both doctors and nurses, and insufficient investment in resources can also contribute to underreporting.^14^ Additionally, the possible inclusion and selection bias might also result in low prevalence rate reported in this study.

The incidences of HAIs and three device-associated infections demonstrated declining trends in both the primary analysis, which utilized data from all 6867 sampled hospitals during 2015–2019, and the sub-analysis, which used data from the 476 hospitals that participated throughout the entire study period. Although these findings appear to be associated with improvements in healthcare safety in Chinese hospitals, they should be interpreted cautiously because a decrease in the bacterial culture rate was also observed, and case finding relied on microbiological results. Our study estimated a prevalence rate of overall HAIs by 2.6% in 2015 and 2.2% in 2019, and showed an average incidence of HAIs by 2.3% with a reduction of the incidence rate by 15.4% from 2015 to 2019, which was align closely with a recent academic report in 2025 academic conference on HAIs in China (2.32 in 2016, 1.98 in 2018 and 1.86 in 2020, detail in Supplementary Figure S5). It was also comparable with the data in a large hospital of the United States (16.0% reduction during 2011–2015).^36^ The PPSs also reported a continuous reduction of HAI prevalence in China (3.6% in 2010, 3.2% in 2012, and 2.7% in 2014) since the 2010s.^14^, ^15^, ^16^ The decreased trend of HAIs likely indicated a beneficial effect of the efforts on preventing the HAI with a possible improvement of quality of infection control (Fig. 1, Supplementary Table S1).^15^^,^^16^ However, the clinical relevance of the absolute reduction in incidence rate of HAIs from 1.2% in 2015 to 1.0% in 2019 should be interpreted with caution, as slight trends may achieve statistical significance due to large sample sizes. This factor warrants careful consideration in the ongoing assessment of HAI prevention strategies in China. Some key interventions, including the national action plans, electronic surveillance, and education and training, may have contributed to the observed reduction (Supplementary Table S1).

Our study observed high detection rates of CRAB, followed by MRSA, CRPA and CRKP. The proportions and trends of MDROs in this study were consistent with data from China Antimicrobial Resistance Surveillance System (CRASS) and China Antimicrobial Surveillance Network (CHINET).^37^^,^^38^ The CRAB and CRKP are the main HAI-associated problems in China.^39^ The CRAB detection rate consistently ranked among the highest at about 50.0% every year, which was very close to that of another domestic study at 51.0% and slightly lower than that in Europe (61.0%) and Southeast Asia (62.0%).^40^^,^^41^ We found a decline trend of CRAB detection, however, the results showed that the detection rate of CRAB remained relatively stable in 2018 and 2019 after a brief drop from 2015 to 2017, leading to the insignificance of the 5-year downward trend. The CRKP detection rate even kept a upward trend, which was similar to the conclusion of one study conducted in Saudi Arabia.^28^ In China, the dominant genotypes of CRKP were *K. pneumoniae* carbapenemase-2 (KPC-2) and the dominant clone of CRKP is sequence type (ST) 11, of which the spread may contribute to the increasing trend of CRKP infection.^42^ Moreover, despite the detection rate of MRSA ranked the second in most regions of China according to our results, an annual decline had been identified, which was similar to the other study performed on MRSA-infections among all nosocomial *S. aureus*-infections in Germany between 2007 and 2016.^43^ The detection rates of VREfm and VREfs in our study were lower than those in other countries, which may be partially related to the infrequent use of vancomycin oral preparations.^37^ Although the incidence of HAIs may be relatively low or even declining, the increasing trend of MDROs highlights the severity of drug resistance of clinical infectious pathogens and continues to pose a public health challenge that requires sustained attention.

In 2016–2017, China's NHC had promulgated some national actions for HAI prevention, such as: guidelines for HAI outbreak control, guidelines for training of HAI management, which appears to have had a positive impact on HAI prevention. Considering the lag effect of new standards, we compared the change of QIs of HAI between 2015‒2017 and 2018–2019 (Supplementary Table S6). The incidence rate of HAI decreased after the promulgation of these standards, which was in consistent with the trend observed nationwide as well as within each subgroup (Table 1, Fig. 4). However, the change of some species of MDROs caused by the standards implemented in 2017 revealed an apparent divergence with the national trend, e.g., CRAB, CRPA, MRSA and VREfs. Although the implementation of 12 new standards in 2017 may have contributed to a decline in the incidence rate of HAI and the detection rates of certain MDROs, it is noteworthy that the detection rates of CRAB in non-tertiary hospitals, as well as those of CRAB, CRPA in hospitals located in developed regions, still exhibit an increasing trend, therefore warranting closer attention (Table 1, Fig. 4). Furthermore, given the increasing detection rates of CRKP across nearly all subgroups, healthcare facilities should strengthen targeted prevention and control measures for this pathogen. Notably, the apparent discrepancy between the declining national incidence of HAIs and the increasing detection rates of certain MDROs, such as CRKP, requires further investigation. As shown in Table 1, the overall reduction in HAI incidence was primarily driven by significant decreases in certain infection types, including CAUTI and VAP. In contrast, MDRO detection rates reflect the antimicrobial resistance profiles of circulating pathogens, which are shaped by distinct factors such as clonal expansion and antimicrobial selection pressure.^39^ Although detection rates of some MDROs, such as CREC, MRSA, and VREfm, have significantly decreased, the detection rates of some MDROs remains high, with CRAB, CRPA, MRSA, and CRKP all exceeding 10%, indicating ongoing transmission within healthcare settings. The high detection rate of CRKP may be driven by plasmids harboring resistance genes such as KPC-2, as well as the predominance of successful clones, particularly sequence type 11, in China.^44^ Furthermore, the increased use of carbapenems may selectively favor CRKP emergence even in the context of improving overall infection prevention and control (IPC).^45^^,^^46^ This pattern, where general IPC success coexists with the rise of a specific, highly adaptable MDRO, highlights the necessity of complementing broad IPC strategies with targeted interventions, including active surveillance and enhanced contact precautions for high-risk pathogens such as CRKP.^44^^,^^47^

The spatiotemporal heterogeneity of HAIs was also observed in our study. The HAIs and MDROs in southeast were higher than the west and northeast, the regional distributions of MDROs were consist with the results from CRASS and CHINET.^37^ The spatiotemporal heterogeneity may be explained by several possibilities such as the uneven distribution of regional medical resources, the difference in the staffing levels per se and the experience of medical staff and the divergence in the attention of local prevention and control policies as well as the potential distribution bias of tertiary and non-tertiary care hospitals across provinces in this study.^14^ First, the distinct microbiological characteristics of certain MDROs are important factors in their transmission. For example, the resistance genes of CRKP are typically located on plasmids,^44^ allowing for rapid transfer between different strains, while MRSA primarily spreads through clonal expansion, with a less efficient transmission mechanism.^48^ Second, the implementation of antimicrobial stewardship has impacted the trends in MDROs. In recent years, China has restricted the use of broad-spectrum β-lactams, including third-generation cephalosporins, particularly for prophylactic purposes. While this may have reduced the selective pressure on MRSA, carbapenem use has increased concurrently, likely due to the rising incidence of infections caused by carbapenem-resistant Gram-negative bacteria (e.g., CRKP and CRAB), for which carbapenems remain a key therapeutic option in severe cases. This trend highlights a practical challenge in antimicrobial stewardship, where clinical demands for effective treatments against multidrug-resistant pathogens may partially offset stewardship efforts. Third, infection prevention and control measures have shown varying effectiveness against different MDROs. Hand hygiene, isolation, and other standard infection prevention measures have shown some effects on MRSA, with the MRSA rate decreasing in some hospitals.^46^^,^^49^ In contrast, CRAB can survive for weeks in hospital environments and may not be completely eradicated through routine cleaning.^50^ A Chinese antimicrobial resistance study also indicated that higher prevalence of antibiotic resistance was also associated with increased regional ambient temperature, higher antibiotic consumption and higher density of hospital beds.^47^

The reasons for the observed spatiotemporal heterogeneity in device-associated infections may be explained as follows: First, the implementation of infection prevention and control measures. Hand hygiene practices and infection prevention bundles are implemented differently across provinces.^51^ Regions that have seen a reduction in incidence trends likely have stricter adherence to infection control measures.^52^^,^^53^ Second, the surveillance of device-related HAIs also plays a role. Differences in subjective assessments and laboratory capabilities can result in varying levels of HAI surveillance, which may contribute to regional discrepancies in device-associated infections.^35^ Currently, China is advocating for an increase bacterial culture rate in the pre-use of therapeutic antibiotics to improve the effectiveness of precision treatment and the diagnosis of HAI. This study found that the bacterial culture rate in some provinces has declined, highlighting the need to focus on strengthening prevention and control efforts in this area.

This study has several limitations. First, while we collected a large volume of data throughout the research, we upheld rigorous standards for data cleaning (see Supplementary Methods) to ensure its accuracy. Any data with potential quality concerns were excluded from the final calculations of the indicators. Therefore, the number of eligible hospitals for some QIs analysis in individual provinces was small with unrepresentative results in some areas (e.g., Tibet of southwestern China). Second, some indicators were not included in the analysis due to the missing data, resulting in the lack of some information such as the underreporting rate of HAIs, which is helpful for adjusting the estimate of burden of HAIs. Third, in the calculation of different QIs as well as the trend analysis, the number of eligible hospitals varied across years. Notably, the participation of non-tertiary care and smaller hospitals increased over time, which may impact the comparability of results and potentially introduce selection bias. The sub-analysis conducted exclusively using data from the 476 hospitals that participated throughout all study years revealed slightly differing trends for QIs compared to the primary analysis, although most of results remained consistent with the primary analysis findings. Fourth, the significant increase in admissions within the 476-hospital subgroup may reflect hospital expansion, which aligns with broader national trends. However, our dataset did not provide sufficient granularity to fully capture this specific dynamic. Fifth, the variation in 12 new standards compliance across regions may contribute to the spatial heterogeneity of HAI/MDRO observed in this study, but it is difficult to accurately estimate compliance in different areas. Sixth, this study does not examine the impact of advancements and disparities in surveillance efforts on regional variations in HAI rates (e.g., between southeastern coastal provinces and inland provinces), which presents a promising avenue for future research. However, our study explored the temporal and spatial patterns and dynamics of the burden of HAIs and detection of MDROs based on the nation-wide longitudinal surveillance data, and highlighted high-risk areas of HAIs and MDROs of concern. Improved surveillance and data analyses are needed based on individual level of HAIs in the future.

In summary, based on consecutive surveillance data from thousands of hospitals, the endemic burden and spatiotemporal heterogeneity of HAI in China is described as one of the first comprehensive descriptions. As in other countries, the burden of HAI in China needs to be highlighted for the large number of patients affected every year and its impact in terms of excess costs and other complications. Our findings contribute to addressing important gaps of how the burden of HAIs and detection of MDROs change in temporal and spatial dimensions across the nation-wide scale of China. The evidence-based health measures could include focusing on hospitals located in higher income region with more beds, especially those located in south China, and by improving the surveillance and control of MDROs with higher detection.

## Contributors

WL, L-QF, and Y-XL conceived, designed and supervised the study. H-WY, C-LL, YT, Z-HY, M-MD, C-XM, A-RZ, B-GJ, QX and J-JC collected, cleaned and analyzed the data. H-WY, C-LL, YT, Y-ZZ, M-MD, J-JS, SZ, YZ, Y-BX, Y-LB, B-WL, Z-QY, J-YL, MC, RH, JL, C-PC, L-QF and WL wrote the draft of the manuscript and interpreted the findings. L-QF, WL, Y-XL, Q-BL and YY commented on and revised drafts of the manuscript. All authors read and approved the final report.

## Data sharing statement

The datasets used and/or analyzed during the current study are available from the corresponding author on reasonable request.

## Editor note

The Lancet Group takes a neutral position with respect to territorial claims in published maps and institutional affiliations.

## Declaration of interests

The authors declare that they have no competing interests.

## Appendix A Supplementary data

Supplemental Data (Clean)

Translated Abstract Final
