# Supplementary material for: Mapping the landscape of healthcare-associated infections in China, 2015–2019: a nation-wide observational study
Source: Lancet Reg Health West Pac. 2025 Dec 16;65:101775. doi: 10.1016/j.lanwpc.2025.101775 (PMC12768928; doi:10.1016/j.lanwpc.2025.101775)

**Supplemental Data:**

Yao HW, et al. Mapping the landscape of healthcare-associated infections in China, 2015−2019: A nation-wide observational study

**Table of Contents**

| **Page** | **Item** |
| --- | --- |
| 2 | Supplementary methods |
| 3 | Table S1. Annual events for the prevention and control for healthcare-associated infection (HAI) in China from 1986 to 2019. |
| 5 | Table S2. Definition and calculation of the key quality indicators of healthcare-associated infection (HAI). |
| 6 | Figure S1. A flowchart of data sorting and cleaning procedures. |
| 7 | Figure S2. The spatial grouping of provinces into North China (blue) and South China (red). |
| 8 | Table S3. Definitions of healthcare-associated infections between US Centers for Disease Prevention and Control and the Ministry of Health of the People’s Republic of China, as referenced in the literature. |
| 19 | Table S4. Mann-Kendall test for the annual trends of healthcare-associated infection (HAI) quality indicators. |
| 21 | Table S5. Sub-analysis for the annual trends of healthcare-associated infection (HAI) quality indicators by exclusively incorporating data from the 476 hospitals in China that participated throughout the entire study period from 2015 to 2019. |
| 24 | Figure S3. The annual change of three device-associated infection rates in different provinces from 2015 to 2019. |
| 25 | Table S6. Comparison of healthcare-associated infection (HAI) indicators before and after the implementation of the new standards for HAI. |
| 26 | Figure S4. Comparison of annual trend of HAI-related quality indicators (QIs) between different subgroups after adjustment for multiple comparison. |
| 27 | Table S7. The factors associated with the detection rate of carbapenem-resistant *Acinetobacter baumannii* (CRAB) in China, 2015-2019. |
| 28 | Table S8. The factors associated with the detection rate of carbapenem-resistant *Escherichia coli* (CREC) in China, 2015-2019. |
| 29 | Table S9. The factors associated with the detection rate of carbapenem-resistant *Klebsiella pneumonia* (CRKP) in China, 2015-2019. |
| 30 | Table S10. The factors associated with the detection rate of carbapenem-resistant *Pseudomonas aeruginosa* (CRPA) in China, 2015-2019. |
| 31 | Table S11. The factors associated with the detection rate of methicillin-resistant *Staphylococcus aureus* (MRSA) in China, 2015-2019. |
| 32 | Table S12. The factors associated with the detection rate of vancomycin-resistant *Enterococcus faecalis* (VREfs) in China, 2015-2019. |
| 33 | Table S13. The factors associated with the detection rate of vancomycin-resistant *Enterococcus faecium* (VREfm) in China, 2015-2019. |
| 34 | Table S14. The factors associated with the incidence density of central line-associated bloodstream infection (CLABSI) in China, 2015-2019. |
| 35 | Table S15. The factors associated with the incidence density of catheter-associated urinary tract infection (CAUTI) in China, 2015-2019. |
| 36 | Table S16. The factors associated with the incidence density of ventilator-associated pneumonia (VAP) in China, 2015-2019. |
| 37 | Figure S5. Trend of current point prevalence survey from 2001 to 2024. |

**Supplementary methods**

**Data collection and sorting**

During the second half of each year, the NHC of China requires the provincial NHCs to guide enrolled hospitals to fill in data of key quality indicators (QIs) of HAIs through the surveillance system of NNIMQCC according to the technical guidance documents. The number of sampled hospitals varies among different provinces, and the sampled hospitals in each province must cover hospitals at all levels including general and specialized hospitals, tertiary and non-tertiary hospitals. After five-year running, the sampled hospitals ranged from 2298 in 2015 to 12,292 in 2019 in China had reported their surveillance data of key QIs of HAIs for aggregation into a national database.

The yearly data were sorted and cleaned following standard criteria and procedures: At the hospital level, those with missing or incomplete data for over six months in each annual surveillance cycle were excluded; those with overall incidence rate of HAI exceeding 50.0% were considered as outlier and excluded. Each record of the data was reviewed by cross checking of two full time staffs in the Department of Infection Management and Disease Control, and differences and assessment were resolved by consensus. Finally, a clean dataset was identified and used for nation-wide analysis in this study, of which the total numbers of eligible hospitals varied from 2773 (number of eligible hospitals for detection of VREfm) to 8652 (number of eligible hospitals for inpatient antimicrobial usage).

**The collection of hospital baseline data**

For each hospital, the baseline information including locations of hospitals (urban or rural areas, developed or developing regions, which was defined by using the World Bank's classification Standard of gross domestic product (GDP) per capita in 2020 (www.worldbank.org), hospital level (non-tertiary, tertiary), total number of beds, and annual number of inpatients, were collected from the reporting system of NNIMQCC. Furthermore, hospitals distributed in urban/developed or rural/developing regions were based on the city or county level, respectively. GDP per capita at province level was collected from the National Bureau of Statistics of China. The 12 new specialized standards targeted for HAI prevention and control were officially released in the late 2016 and implemented by NHC in June 2017 ([www.nhc.gov.cn](http://www.nhc.gov.cn)), which was regarded as an important time point of HAI related major events during the study period from 2015 to 2019, which defined as an important intervention measures in our study, These interventional guidelines included HAI outbreak control, training of HAI management, standardization for sterilization techniques, etc (Supplementary Figure S1, Supplementary Table S1). Timelines of major events for HAI in China from 1986 to 2019 were collected from governmental websites.

**Generalized estimation equation**

Univariate generalized estimation equation (GEE) analysis was conducted to determine the variables which would be included in the multivariate GEE analysis. We considered up to the square orders for all the continuous variables. The polynomial order was chosen by examining the p values. If a square order met the inclusion criteria (p values < 0.2), then its first order was also kept. Multivariate analysis was initiated by including all variables (continuous variables with the polynomial order and categorical variables) with p values < 0.2 in the univariate analysis. We first used correlation analysis to identify whether there was collinearity among the variables. If a relatively high correlation between any two variables is observed (the correlation coefficient is more than 0.6), the one with larger QIC is then dropped. We then used a backward selection procedure to eliminate variables and QIC statistic to help choosing optimal model. At each step, all variables with p values > 0.05 form a potential removal set. For each continuous variable, only the square orders term is included in this potential removal set. Among all the variables that remain in the set, the one can be removed if its removal leads to the largest reduction in the QIC statistic, and the procedure moves to the next step. This procedure combines QIC with p-value, balancing between parsimony and statistical significance. It also removes square orders before removing first orders, ensuring interpretability of the model. The final optimal model obtained by this procedure only retains the variables with p values < 0.05.

**Table S1. Annual events for the prevention and control for healthcare-associated infection (HAI) in China from 1986 to 2019.**

| **Year** | **Events** |
| --- | --- |
| 1986 | National HAI management group and monitoring network were established. |
| 1988 | "Interim Measures for Establishment and Improvement of Nosocomial Infection Management Organization" was promulgated. |
| 1989 | National training base of HAI was established and "Nosocomial Infection and Management" was published. |
| 1990 | The first book of "Nosocomial Infection" was published in China. |
| 1991 | "Chinese Journal of Nosocomiology" started publication and "Measures for the Implementation of the Law of the People's Republic of China on the Prevention and Treatment of Infectious Diseases" was promulgated. |
| 1992 | HAI control branch of Chinese Preventive Medicine Association was established. |
| 1994 | "Emergency Notice on Further Strengthening the Management of Nosocomial Infection" was promulgated. The first provincial quality control center of HAI was established, and "Guidelines for Management of Nosocomial Infections (for trial implementation)" was promulgated. |
| 1995 | "Hygienic Standard of Hospital Disinfection" and other relevant regulations were promulgated. |
| 1997 | National training course on hospital waste management was jointly held by Ministry of Public Health and World Health Organization. |
| 1998 | The national training base and monitoring network of HAI were integrated. |
| 1999 | A seminar on HAI management was held. |
| 2000 | "Guidelines for Management of Nosocomial Infections (for trial implementation)" was revised. |
| 2001 | "Diagnostic Criteria of Nosocomial Infection" was promulgated and the national prevalence survey was carried out. |
| 2002 | "Standardization for Sterilization Techniques" was promulgated and "Chinese Journal of Infection Control" started publication. |
| 2004 | "National Guidance on Health-Care Worker Exposure to HIV (for trial implementation)", "Operating Technique Standard of Cleaning and Disinfection for Endoscope", "Guiding Principles of Clinical Use of Antibiotics" and other relevant documents were promulgated. |
| 2005 | "Management Measures for Pre-examination Triage of Infectious Diseases in Medical Institutions" and "Standardization for Reuse of Blood Dialyzer" were promulgated. |
| 2006 | "Guidelines for Management of Nosocomial Infections" was promulgated and Nosocomial Infection Control Standards Committee was established. |
| 2008 | "Notice on Strengthening Nosocomial Infection Control of Multi-Drug Resistant Organisms" was promulgated. |
| 2009 | The industry standard of HAI management was promulgated for the first time in China. "Management Standard for Outbreak Report and Disposal of Nosocomial Infection" was promulgated. |
| 2010 | "Notice on Strengthening the Prevention and Control of Non-tuberculosis Mycobacteria Nosocomial Infection", "Technical Guidance for Prevention and Control of Surgical Site Infection (for trial implementation)", "Management Measures for Full-time Personnel of Nosocomial Infection" and other documents were promulgated. |
| 2011 | "Technical Guidance for Prevention and Control of Multidrug-resistant Bacteria Nosocomial Infection" and "Notice on the National Special Rectification Activities of Clinical Application of Antibiotics" were promulgated. |
| 2012 | "Standardization for Sterilization Techniques of medical institutions" was promulgated. |
| 2013 | National Center for Nosocomial Infection Quality Management and Control was established. "Basic Requirements of Nosocomial Infection Management in Primary Medical Institutions" was promulgated and informationized monitoring of HAI started. |
| 2014 | "Regulations on Hygiene and Safety Evaluation of Disinfection Products" was promulgated and informationized monitoring of HAI was carried forward. |
| 2015 | "Quality Control Index of Nosocomial Infection Management (2015)" was promulgated and pilot program for informationized monitoring of HAI was developed. |
| 2017 | 12 new specialized standards targeted for HAI prevention and control issued in 2016 and implemented in 2017 and Notice on Further Strengthening the Management of Clinical Application of Antimicrobial Agents to Contain Bacterial Antibiotics Resistance.  12 new specialized standards:   1. Guideline for professional training about managing of healthcare associated infections (WS/T 525—2016) 2. Central sterile supply department(CSSD)——Part 1:Management standard (WS 310.1-2016) 3. Central sterile supply department(CSSD)——Part 2 :Standard for operating procedure of cleaning,disinfection and sterilization (WS 310.2-2016) 4. Central sterile supply department(CSSD)——Part 2 :Standard for operating procedure of cleaning,disinfection and sterilization (WS 310.2-2016) 5. Regulation for healthcare associated infection control in ward in healthcare facilities (WS/T 510—2016) 6. Regulation for prevention and control of healthcare associated infection of airborne transmission disease in healthcare facilities (WS/T 511—2016) 7. Regulation for disinfection and sterilization technique of dental instruments (WS/T 506—2016) 8. Regulation for cleaning and disinfection technique of flexible endoscope (WS/T 507—2016) 9. Regulation for cleaning and disinfection management of environmental surface in healthcare (WS/T 512—2016) 10. Guideline of control of healthcare associated infection outbreak (WS/T 524—2016) 11. Regulation for washing and disinfection technique of meclical textiles in healthcare facilities (WS/T 508—2016) 12. Regulation for prevention and control of healthcare associated infection in intensive care unit(WS/T 509—2016) |
| 2018 | "Management Standard of Nosocomial Infection in Outpatient and Emergency Department of Medical Institutions" and "Assessment Standard for Prevention and Control of Nosocomial Infection" were implemented. Expert Committee of Quality Control Center of National Nosocomial Infection Management was established. |
| 2019 | Ten fundamental institutions of infection prevention and control in medical institutions were promulgated by National Health Commission of the People’s Republic of China. |

**Table S2. Definition and calculation of the key quality indicators of healthcare-associated infection (HAI).**

| **Indicator** | **Definition** | **Calculation** |
| --- | --- | --- |
| Incidence rate of HAI | The number of new patients acquiring an infection per 100 inpatients followed up for a year in corresponding hospitals | $\frac{\text{number of }\text{new}\text{ HAI}\text{s}}{\text{number of inpatient}\text{s }\text{in corresponding hospitals }\text{during the same period}}\times100\%$ |
| Detection rate of MDRO infection | The positive number of each designated MDRO in 100 bacterium detections in the designated pathogen, and the MDROs include CRAB, CREC, CRKP, CRPA, MRSA, VREfs and VREfm | $\frac{\text{number of }\text{each designated antimicrobial}\text{-resistant bacter}\text{ium}\text{ }\text{detection}}{\text{the total number of isolates for the corresponding bacterial species.}}\times100\%$ |
| Incidence rate of SSI in patients with class I/Clean wounds | The proportion of SSI in patients with class I/Clean wounds | $\frac{\text{number of }\text{ }\text{SSI in patients with class I/Clean woundss}}{\text{number of }\text{surgery}\text{ with class I/Clean wounds }\text{during the same period}}\times100\%$ |
| Incidence density of CLABSI | The proportion of new CLABSI among total days of inpatients using central intravascular catheter during the same period | $\frac{\text{number of }\text{new}\text{ }\text{CLABSI}}{\text{total days of inpatients using central intravascular catheter }\text{during the same period}}\times1000‰$ |
| Incidence density of CAUTI | The proportion of new CAUTI among total days of inpatients using catheter during the same period | $\frac{\text{number of }\text{new}\text{ }\text{CAUTI}}{\text{total days of inpatients using catheter }\text{during the same period}}\times1000‰$ |
| Incidence density of VAP | The proportion of new VAP among total days of inpatients using ventilator during the same period | $\frac{\text{number of }\text{new}\text{ }\text{VAP}}{\text{total days of inpatients using ventilator }\text{during the same period}}\times1000‰$ |
| Inpatient antimicrobial usage rate | The proportion of inpatients antimicrobial usage | $\frac{\text{number of inpatients receiving }\text{antimicrobial treatment}\text{ }}{\text{number of inpatients during the same period}}\times100\%$ |
| Bacterial culture rate | The proportion of bacterial culture among those with therapeutic use of antimicrobial agents | $\frac{\text{n}\text{umber having bacterial cultures before antimicrobial treatment started}}{\text{number of inpatients receiving antimicrobial treatment}\text{ }}\times100\%$ |

QIs: Quality Indicators; HAI: healthcare-associated infection; MDROs: Multi-Drug Resistant Organisms; CRAB: carbapenem-resistant *Acinetobacter baumannii*; CREC: carbapenem-resistant *Escherichia coli*; CRKP: carbapenem-resistant *Klebsiella pneumonia*; CRPA: carbapenem-resistant *Pseudomonas aeruginosa*; MRSA: methicillin-resistant *Staphylococcus aureus*; VREfs: vancomycin-resistant *Enterococcus faecalis*; VREfm: vancomycin-resistant *Enterococcus faecium*; SSI: surgical site infections; CLABSI: central line-associated bloodstream infection; CAUTI: catheter-associated urinary tract infection; VAP: ventilator-associated pneumonia.

**Figure S1.** **A flowchart of data sorting and cleaning procedures.** 40,373 represents the total number of data packets (records) submitted by all participating hospitals throughout the study period. The subsequent figures indicate the number of data packets retained or excluded at each stage of data cleaning for each respective index. Each data packet includes both the numerator and denominator values for specific HAI quality control indicators reported by individual hospitals in given years, such as the “number of new HAIs” and the “number of inpatients”.


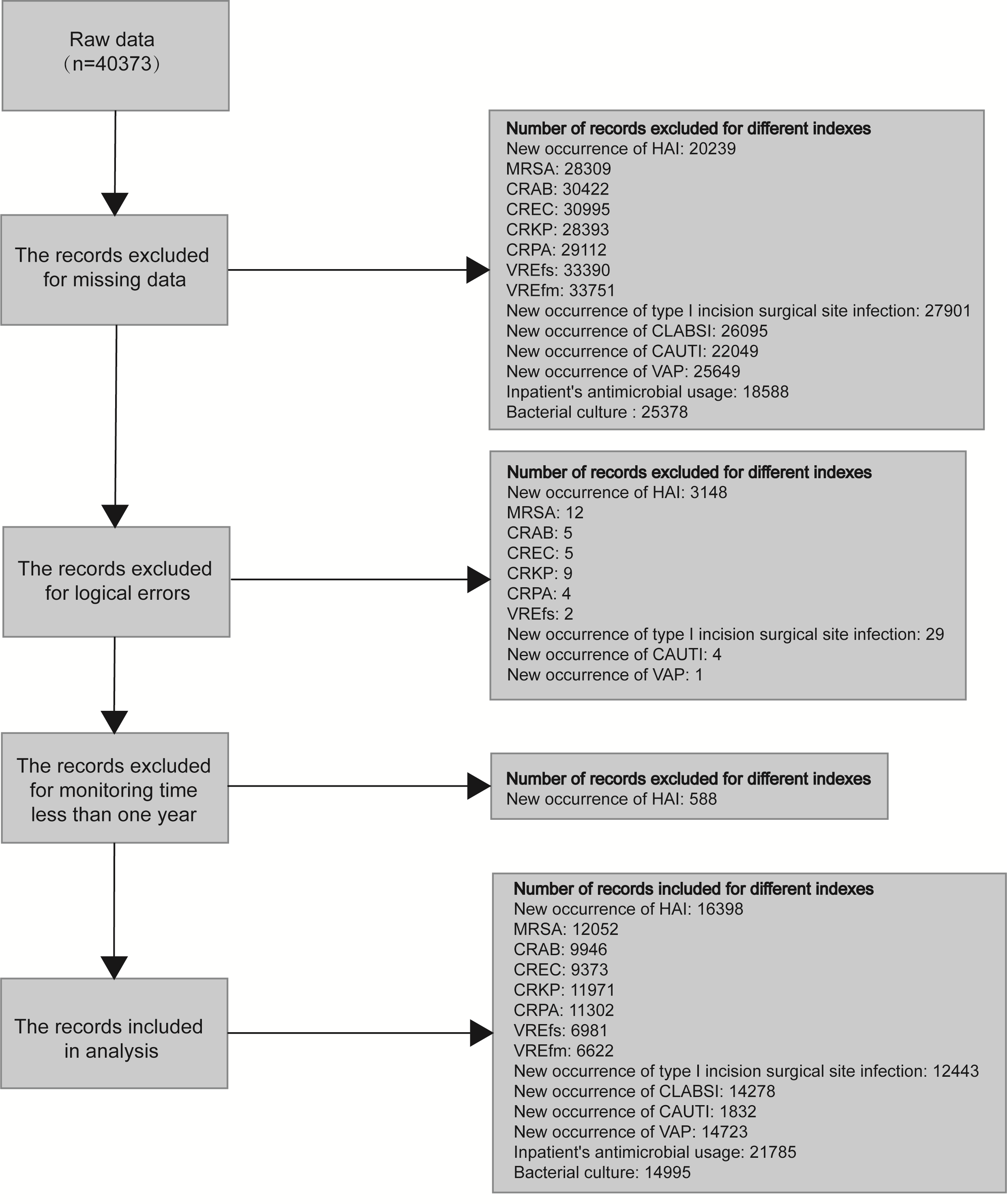


**Figure S2. The spatial grouping of provinces into North China (blue) and South China (red).**


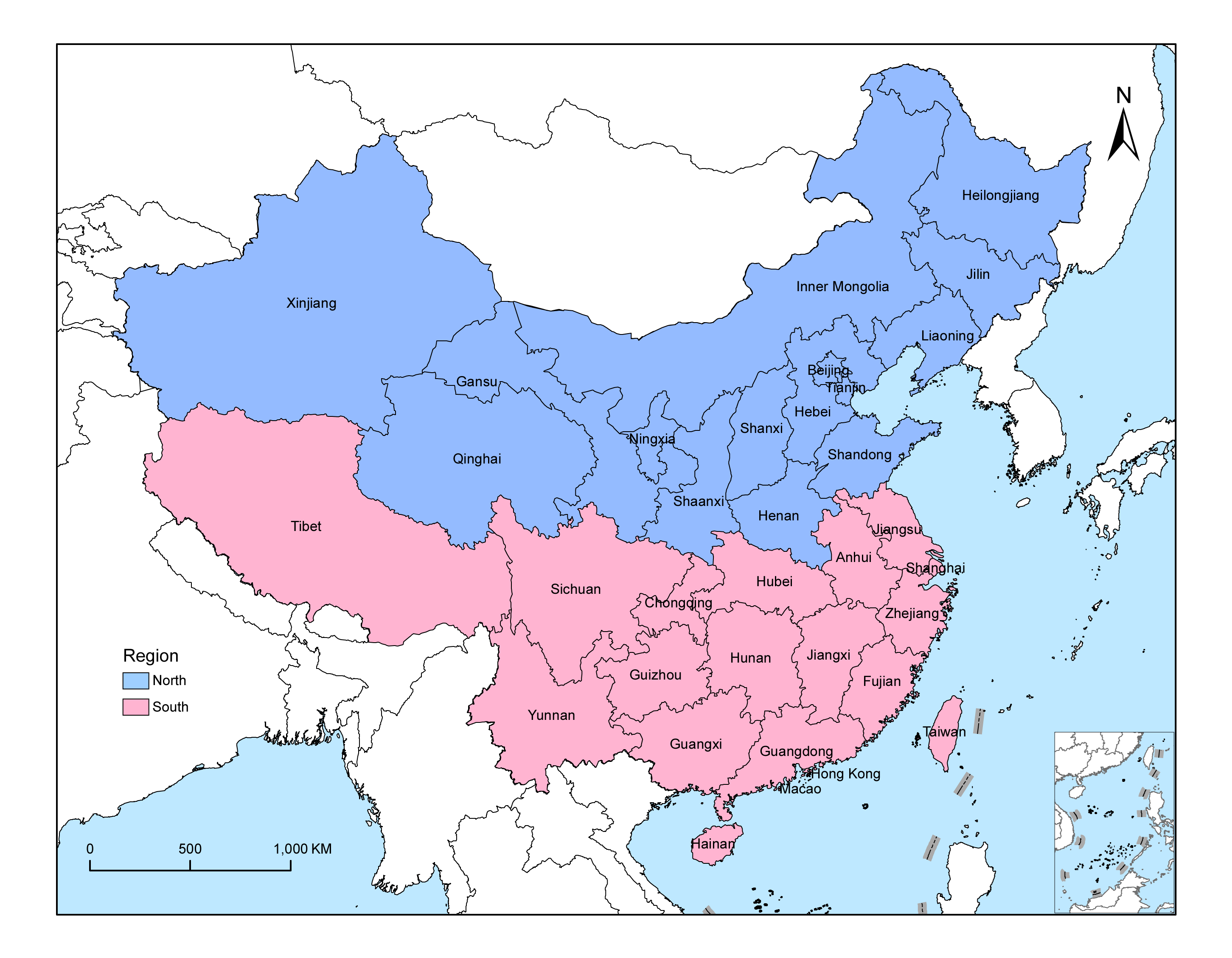


**Table S3. Definitions of healthcare-associated infections between US Centers for Disease Prevention and Control and the Ministry of Health of the People’s Republic of China, as referenced in the literature** (*Wang J, Hu J, Harbarth S, Pittet D, Zhou M, Zingg W. Burden of healthcare-associated infections in China: Results from the 2015 point prevalence survey in Dongguan City. J Hosp Infect. 2017;96(2):132-138)****.***

|  | **US Centers for Disease Prevention and Control** | **Ministry of health of the People’s Republic of China** |
| --- | --- | --- |
| **Urinary tract infection** | 1. At least one of the following: fever, urgency, frequency, dysuria or suprapubic tenderness   **AND**  Patient has a positive urine culture >10^5^ micro-organisms per cc of urine   1. At least two of the following: fever, urgency, frequency, dysuria or suprapubic tenderness   **AND**  At least one of the following:   1. Positive dipstick for leucocyte esterase 2. Urine specimen with >10 white blood cells 3. Organisms seen on Gram’s stain of unspun urine 4. At least two urine cultures with repeated isolation of the same uropathogen 5. <10^5^ colonies/mL of a single uropathogen (Gram-negative bacteria or *S. saprophyticus*) 6. Physician diagnosis of a urinary tract infection 7. Physician institutes appropriate therapy for a urinary tract infection | Frequency, urgency, dysuria OR suprapubic tenderness  **AND**  One of following:   1. Urine specimen with ≥5 white blood cell high power field (male) 2. >10 white blood cell high power field (female) 3. Urinary culture positive if patient with urinary catheter 4. Physician diagnosis of a urinary tract infection   **WITH or WITHOUT**  One of following:   1. Gram-positive cocci ≥10^4^ 2. Gram negative bacilli ≥10^5^ 3. Suprapubic puncture ≥10^3^ bacteria 4. Centrifugal urinary specimens by phase contrast microscopy (×400): identification of bacteria in 50% of 30 microscopy fields |
| **Superficial surgical site infection** | Infection occurs within 30 days after the operative procedure  **AND**  Involves only skin and subcutaneous tissue of the incision  **AND**  Patient has at least one of the following:   1. Purulent drainage from the superficial incision 2. Organisms isolated from an aseptically obtained culture of fluid or tissue from the superficial incision 3. At least one of the following signs or symptoms of infection: pain or tenderness, localized swelling, redness, or heat, AND superficial incision is deliberately opened by surgeon and is culture positive or not cultured (A culture-negative finding does not meet this criterion) 4. Diagnosis of superficial incisional surgical site infection by the surgeon or attending physician   Comments:   1. Do not report a stitch abscess (minimal inflammation and discharge confined to the points of suture penetration) as an infection 2. Do not report a localized stab wound infection as SSI, instead report an skin, or soft tissue, infection, depending on its depth 3. Report infection of the circumcision site in newborns as CIRC 4. Infected burn wound as BURN 5. If the incisional site infection involves or extends into the fascial and muscle layers, report as a deep incisional surgical site infection 6. Classify infection that involves both superficial and deep incision sites as deep incisional surgical site infection | Infection occurs within 30 days after the operative procedure  **AND**  Involves only skin and subsutaneous tissue of the incision  **AND**  Patient has at least one of the following:  a. Redness, localized swelling, heat, and pain or tenderness, or purulent discharge  b. Diagnosis of superficial incisional surgical site infection by the surgeon or attending physician  **WITH or WITHOUT**  A positive microbiological culture  Comments:   1. Do not report a stitch abscess 2. Do not report a localized stab wound infection as surgical site infection, instead report an skin, or soft tissue, infection, depending on its depth 3. Do not report as surgical site infection, if fat liquefaction only |
| **Deep incisional surgical site infection** | Infection occurs within 30 days after the operative procedure if no implant is left in place or within one year if implant is in place and infection appears to be related to the operative procedure  **AND**  Involves deep soft tissues of the incision  **AND**  Patient has at least one of the following:   1. Purulent drainage from the deep incision but not from the organ/space component of the surgical site 2. A deep incision spontaneously dehisces or is deliberately opened by a surgeon and is culture-positive or not cultured when the patient has at least one of the following signs or symptoms: fever (38°C), or localized pain or tenderness (a culture negative finding does not meet this criterion) 3. An abscess or other evidence of infection involving the deep incision is found on direct examination, during reoperation, or by histopathologic or radiologic examination 4. Diagnosis of a deep incisional surgical site infection by a surgeon or attending physician   Comments:  Classify infection that involves both superficial and deep incision sites as deep incisional surgical site infection | Infection occurs within 30 days after the operative procedure if no implant is left in place or within one year if implant is in place and infection appears to be related to the operative procedure  **AND**  Involves deep soft tissues of the incision  **AND**  One of following:   1. ‘Purulent drainage from deep incision’ OR ‘puncture purulent’ 2. A deep incision spontaneously dehisces or is deliberately opened by a surgeon and is culture-positive, or an abscess, or fever, or localized pain or tenderness 3. An abscess or other evidence of infection involving the deep incision is found on direct examination, during reoperation, or by histopathologic or radiologic examination 4. Diagnosis of a deep incisional surgical site infection by a surgeon or attending physician   **WITH or WITHOUT**  A positive microbiological culture |
| **Organ/space surgical site infection** | Infection occurs within 30 days after the operative procedure if no implant is left in place or within one year if implant is in place and the infection appears to be related to the operative procedure  **AND**  Infection involves any part of the body, excluding the skin incision, fascia, or muscle layers, that is opened or manipulated during the operative procedure  **AND**  Patient has at least one of the following: purulent drainage from a drain that is placed through a stab wound into the organ/space; organisms isolated from an aseptically obtained culture of fluid or tissue in the organ/space; an abscess or other evidence of infection involving the organ/space that is found on direct examination, during reoperation, or by histopathologic or radiologic examination; diagnosis of an organ/space surgical site infection by a surgeon or attending physician  Comments:  Specific sites of organ/space surgical site infection (see also criteria for these sites); Occasionally an organ/space infection drains through the incision. Such infection generally does not involve reoperation and is considered a complication of the incision; therefore, classify it as a deep incisional surgical site infection | Infection occurs within 30 days after the operative procedure if no implant is left in place or within one year if implant is in place and the infection appears to be related to the operative procedure  **AND**  Infection involves any part of the body, excluding the skin incision, fascia, or muscle layers, that is opened or manipulated during the operative procedure  **AND**  One of following:   1. ‘Purulent drainage from incision’ OR ‘puncture purulent’ 2. An abscess or other evidence of infection involving the organ/space incision is found on direct examination, during reoperation, or by histopathologic or radiologic examination 3. Diagnosis of an organ-space surgical site infection by a surgeon or attending physician   **WITH or WITHOUT**  A positive microbiological culture  Comments:   1. Report as surgical site infection if having typical signs and syndromes of organ/space surgical site infection, even though negative bacterial culture 2. Classify it as a deep incisional surgical site infection, if having superficial surgical site infection and deep surgical site infection 3. Occasionally an organ/space infection drains through the incision; such infection generally does not involve reoperation and is considered a complication of the incision; therefore, classify it as a deep incisional surgical site infection |
| **Bloodstream infection/sepsis** | **Laboratory-confirmed bloodstream infection**   1. Patient has a recognized pathogen cultured from one or more blood cultures   **AND**  Organism cultured from blood is not related to an infection at another site   1. Patient has at least one of the following signs or symptoms: fever (>38°C), chills, or hypotension   **AND**  Signs and symptoms and positive laboratory results are not related to an infection at another site  **AND**  Common skin contaminant is cultured from two or more blood cultures drawn on separate occasions | **(Clinical) sepsis**  Body temperature ≥38℃ or <36℃, with/without chills  **AND**  One of the following:   1. Invasive portal or migratory lesions 2. No apparent infection at another site 3. A rash or bleeding or hepatosplenomegaly or elevated neutrophils with left shift AND no other reasons to be explained 4. Systolic blood pressure <12 kPa (90 mmHg) or the baseline systolic blood pressure fell more than 5.3 kPa (40 mmHg)   **WITH or WITHOUT**  One of the following:   1. Positive blood culture 2. Pathogen antigens |
| **Catheter infection** | **Catheter exit-site infection**   1. Patient has at least one of the following signs with no other recognized cause: fever (>38°C), pain, erythema, or heat at involved vascular site   **AND**  More than 15 colonies cultured from intravascular cannula tip using semiquantitative culture method  **AND**  Blood culture not done or no organisms cultured from blood   1. Patient has purulent drainage at involved vascular site   **AND**  Blood culture not done or no organisms cultured from blood | **Catheter-related infection**  At least one of the following:   1. Access site with purulent discharge or diffuse erythema (cellulitis performance) 2. Pain in the subcutaneous catheter route 3. Cardiac catheterization or body temperature ≥38℃ or local tenderness AND no other reasons to link the infection   **AND/OR**  A catheter tip culture, and/or a blood culture to isolate a pathogenic micro-organism.  Comments:   1. Catheter tip culture should examine the distal 5 cm; cut-off: ≥15 cfu/plate is positive 2. Quantitative culture; cut off: bacterial count ≥100 cfu/mL OR same bacteria isolated from contralateral blood cultures and 4–10 times more bacteria are isolated from the affected side |
| **Lower respiratory tract infection** | **Bronchitis, tracheobronchitis, bronchiolitis, tracheitis, without evidence of pneumonia**  Tracheobronchial infections must meet at least one of the following criteria:  Patient has no clinical or radiographic evidence of pneumonia  **AND**  Patient has at least two of the following signs or symptoms with no other recognized cause: fever (>38°C), cough, new or increased sputum production, rhonchi, wheezing  **AND**  At least one of the following:  a. Positive culture obtained by deep tracheal aspirate or bronchoscopy  b. Positive antigen test on respiratory secretions  Reporting instructions:  Do not report chronic bronchitis in a patient with chronic lung disease as an infection unless there is evidence of an acute secondary infection, manifested by change in organism. | **Bronchitis, tracheobronchitis, bronchiolitis, tracheitis, pneumonia**  At least one of the following:  Cough, sputum, rales  **AND**  One of following:   1. Fever (≥38°C), elevated white blood cells, radiographic lesions 2. Acute secondary infection in chronic pulmonary disease AND positive culture changes OR change of radiographic lesions   **WITH or WITHOUT**  At least one of the following:   1. Positive quantitative culture from minimally contaminated lower respiratory tract specimen 2. Bacteria count ≥10^6^ cfu/mL 3. Positive blood culture, AND/OR positive pleural fluid culture 4. Intracellular bacteria on direct microscopic exam in bronchoalveolar lavage 5. Isolation of true pathogens 6. Bacterial antigens in blood or histopathology |
| **Gastrointestinal infection** | Gastrointestinal tract infections, excluding gastroenteritis and appendicitis, must meet at least one of the following criteria:  1. Patient has an abscess or other evidence of infection seen during a surgical operation or histopathologic examination  2. Patient has at least two of the following signs or symptoms with no other recognized cause and compatible with infection of the organ or tissue involved: fever (>38℃), nausea, vomiting, abdominal pain, or tenderness  **AND**  At least one of the following:   1. Organisms cultured from drainage or tissue obtained during a surgical operation or endoscopy or from a surgically placed drain 2. Organisms seen on Gram’s or potassium hydrogen stain or multinucleated giant cells seen on microscopic examination of drainage or tissue obtained during a surgical operation or endoscopy or from a surgically placed drain 3. Organisms cultured from blood 4. Evidence of pathologic findings on radiographic examination 5. Evidence of pathologic findings on endoscopic examination (e.g. candida oesophagitis or proctitis) | Fever (≥38℃), nausea, vomiting  **AND/OR**  Abdominal pain or diarrhoea, no other reasons to explain infection  **WITH or WITHOUT**  At least one of the following:   1. Organisms cultured from drainage (surgical procedure or drain) or tissue obtained by surgical procedure or endoscopy 2. Organisms and multi-nucleated giant cells seen on Gram’s or potassium hydrogen stain 3. Evidence of infection in histopathology |
| **Intra-abdominal infection** | Intra-abdominal infections must meet at least one of the following criteria:   1. Patient has organisms cultured from purulent material from intra-abdominal space obtained during a surgical operation or needle aspiration 2. Patient has abscess or other evidence of intra-abdominal infection seen during a surgical operation or histopathologic examination 3. Patient has at least two of the following signs or symptoms with no other recognized cause: fever (>38℃), nausea, vomiting, abdominal pain, or jaundice   **AND**  At least one of the following:   1. Organisms cultured from drainage from surgically placed drain (e.g. closed suction drainage system, open drain, T-tube drain) 2. Organisms seen on Gram’s stain of drainage or tissue obtained during surgical operation or needle aspiration 3. Organisms cultured from blood and radiographic evidence of infection (e.g. abnormal findings on ultrasound, CT scan, MRI, or radiolabel scans [gallium, technetium, etc.] or on abdominal X-ray)   Reporting instructions:  Do not report pancreatitis (an inflammatory syndrome characterized by abdominal pain, nausea, and vomiting associated with high serum levels of pancreatic enzymes) unless it is determined to be infectious in origin | (Change of) radiological findings  **AND**  Two of following:   1. Fever (≥38℃) 2. Nausea, vomiting 3. Abdominal pain, tenderness 4. Jaundice   **WITH or WITHOUT**  One of the following:   1. Organisms cultured from a surgically placed drain 2. Positive blood culture with the same pathogen as isolated in the local lesion or compatible with the clinical signs |
| **Skin infection** | Skin infections must meet at least one of the following criteria:   1. Patient has purulent drainage, pustules, vesicles, or boils 2. Patient has at least two of the following signs or symptoms with no other recognized cause: pain or tenderness, localized swelling, redness, or heat   **AND**  At least one of the following:   1. Organisms cultured from aspirate or drainage from affected site; if organisms are normal skin flora (i.e. diphtheroids [*Corynebacterium* spp.], *Bacillus* [not *B. anthracis*] spp, *Propionibacterium* spp, coagulase-negative staphylococci [including *S. epidermidis*], viridans group streptococci, *Aerococcus* spp., *Micrococcus* spp.), they must be a pure culture 2. Organisms cultured from blood 3. Positive antigen test performed on infected tissue or blood (e.g. herpes simplex, varicella zoster, *H. influenzae*, *N. meningitidis*) 4. Multi-nucleated giant cells seen on microscopic examination of affected tissue 5. Diagnostic single antibody titre (IgM) or four-fold increase in paired sera (IgG) for pathogen | One of following:   1. Patient has purulent discharge, pustules, vesicles, or boils 2. Signs or symptoms with no other recognized cause: pain or tenderness, localized swelling, redness, or heat   **WITH or WITHOUT**  One of following:   1. Organisms cultured from aspirate or drainage from affected site 2. Detection of antigens |
| **Soft tissue infection** | Soft tissue infections must meet at least one of the following criteria:   1. Patient has organisms cultured from tissue or drainage from affected site 2. Patient has purulent drainage at affected site 3. Patient has an abscess or other evidence of infection seen during a surgical operation or histopathologic examination 4. Patient has at least two of the following signs or symptoms at the affected site with no other recognized cause: localized pain or tenderness, redness, welling, or heat   **AND**  At least one of the following:   1. Organisms cultured from blood 2. Positive antigen test performed on blood or urine (e.g. *H. influenzae*, *S. pneumoniae*, *N. meningitidis*, Group B streptococcus, *Candida* spp.) 3. Diagnostic single antibody titre (IgM) or four-fold increase in paired sera (IgG) for pathogen | One of following:   1. Purulent discharge 2. Patient has an abscess or other evidence of infection identified during a surgical procedure or in histopathology 3. Localized pain or tenderness, redness, swelling, or heat   **WITH or WITHOUT**  One of following:   1. Detection of IgM or four-fold IgG increase in paired sera for a specific pathogen 2. Patient has organisms cultured from tissue or discharge from affected site |

**Table S4. Mann-Kendall test for the annual trends of healthcare-associated infection (HAI) quality indicators.**

|  | **Total** | **2015** | **2016** | **2017** | **2018** | **2019** | **Linear regression**  **p value** | **Mann-Kendall test**  **p value** |
| --- | --- | --- | --- | --- | --- | --- | --- | --- |
| Occurrence of HAI | | |  |  |  |  |  |  |
| Incidence rate of HAIs (%) | 1.1 | 1.2 | 1.1 | 1.1 | 1.0 | 1.0 | 0.011↓ | 0.028↓ |
| Prevalence calculated from Incidence (%) | 2.2 | 2.5 | 2.3 | 2.2 | 2.1 | 2.0 |  |  |
| Detection of CRAB | | |  |  |  |  |  |  |
| Detection rate of CRAB (%) | 51.1 | 52.3 | 51.5 | 50.3 | 50.6 | 50.7 | 0.099 | 0.462 |
| Detection of CREC | | |  |  |  |  |  |  |
| Detection rate of CREC (%) | 5.3 | 7.1 | 5.7 | 5.1 | 4.4 | 4.0 | 0.005↓ | 0.028↓ |
| Detection of CRKP | | |  |  |  |  |  |  |
| Detection rate of CRKP (%) | 12.4 | 10.5 | 11.0 | 12.8 | 13.6 | 14.3 | 0.004↑ | 0.028↑ |
| Detection of CRPA | | |  |  |  |  |  |  |
| Detection rate of CRPA (%) | 22.8 | 22.8 | 21.9 | 23.4 | 23.3 | 22.8 | 0.510 | 0.806 |
| Detection of MRSA | | |  |  |  |  |  |  |
| Detection rate of MRSA (%) | 31.0 | 34.0 | 31.8 | 30.4 | 29.4 | 29.6 | 0.024↓ | 0.086 |
| Detection of VREfs | | |  |  |  |  |  |  |
| Detection rate of VREfs (%) | 1.2 | 1.3 | 1.1 | 1.1 | 1.1 | 1.6 | 0.505 | 0.613 |
| Detection of VREfm | | |  |  |  |  |  |  |
| Detection rate of VREfm (%) | 2.2 | 3.1 | 2.4 | 2.1 | 2.0 | 1.4 | 0.008↓ | 0.028↓ |
| New occurrence of SSI in patients with class I/Clean wounds | | | |  |  |  |  |  |
| Incidence rate (%) | 0.3 | 0.3 | 0.3 | 0.3 | 0.3 | 0.3 | 0.131 | 0.312 |
| New occurrence of CLABSI |  |  |  |  |  |  |  |  |
| Incidence density (events/1000 days) | 0.8 | 1.0 | 0.8 | 0.7 | 0.7 | 0.7 | 0.081 | 0.221 |
| New occurrence of CAUTI |  |  |  |  |  |  |  |  |
| Incidence density (events/1000 days) | 1.6 | 1.8 | 1.8 | 1.6 | 1.5 | 1.3 | 0.007↓ | 0.043↓ |
| New occurrence of VAP |  |  |  |  |  |  |  |  |
| Incidence density (events/1000 days) | 8.2 | 10.8 | 9.4 | 7.8 | 7.4 | 5.8 | 0.002↓ | 0.028↓ |
| Inpatient antimicrobial usage |  |  |  |  |  |  |  |  |
| Inpatient antimicrobial usage rate (%) | 45.5 | 43.5 | 46.5 | 46.7 | 45.9 | 44.9 | 0.683 | 1.000 |
| Bacterial culture before therapeutic use of antimicrobial agents | |  |  |  |  |  |  |  |
| Bacterial culture rate (%) | 41.4 | 45.2 | 42.1 | 40.5 | 38.9 | 40.2 | 0.065 | 0.086 |

**Table S5. Sub-analysis for** **the annual trends of** **healthcare-associated infection (HAI) quality indicators by exclusively incorporating data from the 476 hospitals in China that participated throughout the entire study period from 2015 to 2019.**

|  | **Total** | **2015** | **2016** | **2017** | **2018** | **2019** | **β coefficient of linear trend (95%CI)** | **p value^*^** |  |  |
| --- | --- | --- | --- | --- | --- | --- | --- | --- | --- | --- |
| Occurrence of HAI | | |  |  |  |  |  |  |  |  |
| Number of eligible hospitals | 476 | 476 | 476 | 476 | 476 | 476 |  |  |  |  |
| Number of inpatients | 120,284,611 | 20,614,810 | 22,440,949 | 24,183,627 | 25,564,329 | 27,480,896 |  |  |  |  |
| Number of new HAIs | 1,542,312 | 284,708 | 297,313 | 306,831 | 322,095 | 331,365 |  |  |  |  |
| Incidence rate of HAIs (%) | 1.3 | 1.4 | 1.3 | 1.3 | 1.3 | 1.2 | **-0.04 (-0.06, -0.02)** | **0.004↓** |  |  |
| Average length of hospital stay (days) | 9.3 | 9.6 | 9.4 | 9.3 | 9.3 | 9.1 |  |  |  |  |
| Prevalence calculated from Incidence (%) | 2.8 | 2.9 | 2.9 | 2.8 | 2.8 | 2.7 | **-0.05 (-0.07, -0.04)** | **0.002↓** |  |  |
| Detection of CRAB | | |  |  |  |  |  |  |  |  |
| Number of eligible hospitals | 353 | 353 | 353 | 353 | 353 | 353 |  |  |  |  |
| Number of CRAB isolates | 371,288 | 56,747 | 66,509 | 74,352 | 84,409 | 89,271 |  |  |  |  |
| Detection rate of CRAB (%) | 54.1 | 50.6 | 52.4 | 55.3 | 54.5 | 56.4 | **1.36 (0.74, 1.98)** | **0.023↑** |  |  |
| Detection of CREC | | |  |  |  |  |  |  |  |  |
| Number of eligible hospitals | 427 | 427 | 427 | 427 | 427 | 427 |  |  |  |  |
| number of CREC isolates | 49,244 | 10,904 | 9840 | 8874 | 10,255 | 9371 |  |  |  |  |
| Detection rate of CREC (%) | 3.4 | 4.6 | 3.7 | 3.1 | 3.3 | 2.8 | **-0.40 (-0.59, -0.21)** | **0.026↓** |  |  |
| Detection of CRKP | | |  |  |  |  |  |  |  |  |
| Number of eligible hospitals | 406 | 406 | 406 | 406 | 406 | 406 |  |  |  |  |
| number of CRKP isolates | 166,411 | 17,212 | 22,901 | 31,593 | 43,028 | 51,677 |  |  |  |  |
| Detection rate of CRKP (%) | 14.5 | 9.9 | 11.9 | 14.0 | 16.3 | 17.7 | **2.01 (1.85, 2.16)** | **<0.001↑** |  |  |
| Detection of CRPA | | |  |  |  |  |  |  |  |  |
| Number of eligible hospitals | 402 | 402 | 402 | 402 | 402 | 402 |  |  |  |  |
| Number of CRPA isolates | 219,396 | 33,314 | 37,544 | 43,715 | 49,964 | 54,859 |  |  |  |  |
| Detection rate of CRPA (%) | 25.4 | 23.5 | 24.0 | 25.7 | 25.6 | 27.7 | **0.99 (0.64, 1.34)** | **0.012↑** |  |  |
| Detection of MRSA | | |  |  |  |  |  |  |  |  |
| Number of eligible hospitals | 429 | 429 | 429 | 429 | 429 | 429 |  |  |  |  |
| Number of MRSA isolates | 208,940 | 37,674 | 38,082 | 41,249 | 43,220 | 48,715 |  |  |  |  |
| Detection rate of MRSA (%) | 31.7 | 34.1 | 31.9 | 31.7 | 29.6 | 31.6 | -0.74 (-1.52, -0.04) | 0.159 |  |  |
| Detection of VREfs | | |  |  |  |  |  |  |  |  |
| Number of eligible hospitals | 233 | 233 | 233 | 233 | 233 | 233 |  |  |  |  |
| Number of VREfs isolates | 1472 | 332 | 184 | 456 | 309 | 191 |  |  |  |  |
| Detection rate of VREfs (%) | 1.0 | 1.3 | 0.6 | 1.6 | 0.9 | 0.6 | -0.12 (-0.39, 0.16) | 0.463 |  |  |
| Detection of VREfm | | |  |  |  |  |  |  |  |  |
| Number of eligible hospitals | 236 | 236 | 236 | 236 | 236 | 236 |  |  |  |  |
| Number of VREfm isolates | 3151 | 745 | 605 | 624 | 548 | 629 |  |  |  |  |
| Detection rate of VREfm (%) | 1.8 | 2.8 | 1.9 | 1.8 | 1.4 | 1.4 | **-0.33 (-0.49, -0.18)** | **0.025↓** |  |  |
| New occurrence of SSI in patients with class I/Clean wounds | | | |  |  |  |  |  |  |  |
| Number of eligible hospitals | 450 | 450 | 450 | 450 | 450 | 450 |  |  |  |  |
| Number of SSI in patients with class I/Clean wounds | 57,350 | 7514 | 18,185 | 8622 | 12,594 | 10,435 |  |  |  |  |
| Incidence rate (%) | 0.4 | 0.3 | 0.7 | 0.3 | 0.4 | 0.3 | -0.04 (-0.16, 0.09) | 0.620 |  |  |
| New occurrence of CLABSI |  |  |  |  |  |  |  |  |  |  |
| Number of eligible hospitals | 370 | 370 | 370 | 370 | 370 | 370 |  |  |  |  |
| Number of CLABSI | 14,129 | 2720 | 2771 | 2714 | 3160 | 2764 |  |  |  |  |
| Incidence density (events/1000 days) | 0.7 | 0.9 | 0.8 | 0.7 | 0.6 | 0.4 | **-0.011 (-0.013, -0.009)** | **0.001↓** |  |  |
| New occurrence of CAUTI |  |  |  |  |  |  |  |  |  |  |
| Number of eligible hospitals | 417 | 417 | 417 | 417 | 417 | 417 |  |  |  |  |
| Number of CAUTI | 51,181 | 8665 | 8868 | 10,029 | 11,677 | 11,942 |  |  |  |  |
| Incidence density (events/1000 days) | 1.4 | 1.6 | 1.5 | 1.6 | 1.4 | 1.2 | **-0.010 (-0.016, -0.005)** | **0.036↓** |  |  |
| New occurrence of VAP |  |  |  |  |  |  |  |  |  |  |
| Number of eligible hospitals | 389 | 389 | 389 | 389 | 389 | 389 |  |  |  |  |
| Number of VAP | 56,477 | 11,855 | 11,827 | 10,610 | 11,237 | 10,948 |  |  |  |  |
| Incidence density (events/1000 days) | 7.5 | 9.5 | 8.7 | 7.2 | 6.9 | 6.0 | **-0.09 (-0.11, -0.07)** | **0.003↓** |  |  |
| Inpatient antimicrobial usage |  |  |  |  |  |  |  |  |  |  |
| Number of eligible hospitals | 343 | 343 | 343 | 343 | 343 | 343 |  |  |  |  |
| Number of inpatients with antimicrobial usage | 39,395,179 | 6,682,865 | 7,570,650 | 7,974,302 | 8,326,771 | 8,840,591 |  |  |  |  |
| Inpatient antimicrobial usage rate (%) | 44.2 | 42.9 | 45.6 | 45.0 | 44.3 | 43.2 | -0.08 (-0.90, 0.75) | 0.863 |  |  |
| Bacterial culture before therapeutic use of antimicrobial agents | |  |  |  |  |  |  |  |  |  |
| Number of eligible hospitals | 241 | 241 | 241 | 241 | 241 | 241 |  |  |  |  |
| Number of patients for bacterial culture | 8,133,842 | 1,475,035 | 1,570,024 | 1,603,643 | 1,689,479 | 1,795,661 |  |  |  |  |
| Bacterial culture rate (%) | 42.7 | 47.3 | 44.4 | 41.4 | 40.8 | 40.9 | **-1.63 (-2.47, -0.79)** | **0.032↓** |  |  |

^*^ Linear regression for trends

HAI: healthcare-associated infection; CRAB: carbapenem-resistant *Acinetobacter baumannii*; CREC: carbapenem-resistant *Escherichia coli*; CRKP: carbapenem-resistant *Klebsiella pneumonia*; CRPA: carbapenem-resistant *Pseudomonas aeruginosa*; MRSA: methicillin-resistant *Staphylococcus aureus*; VREfs: vancomycin-resistant *Enterococcus faecalis*; VREfm: vancomycin-resistant *Enterococcus faecium*; SSI: surgical site infections; CLABSI: central line-associated bloodstream infection; CAUTI: catheter-associated urinary tract infection; VAP: ventilator-associated pneumonia.

↑: Significant increasing tendency. ↓: Significant decreasing tendency.

**Figure S3.** **The annual change of three device-associated infection rates in different provinces from 2015 to 2019.** The colors represent the standardized infection rates at the province level. The arrows indicate the changed directions of the infection rate. CLABSI: central line-associated bloodstream infection; CAUTI: catheter-associated urinary tract infection; VAP: ventilator-associated pneumonia.

**
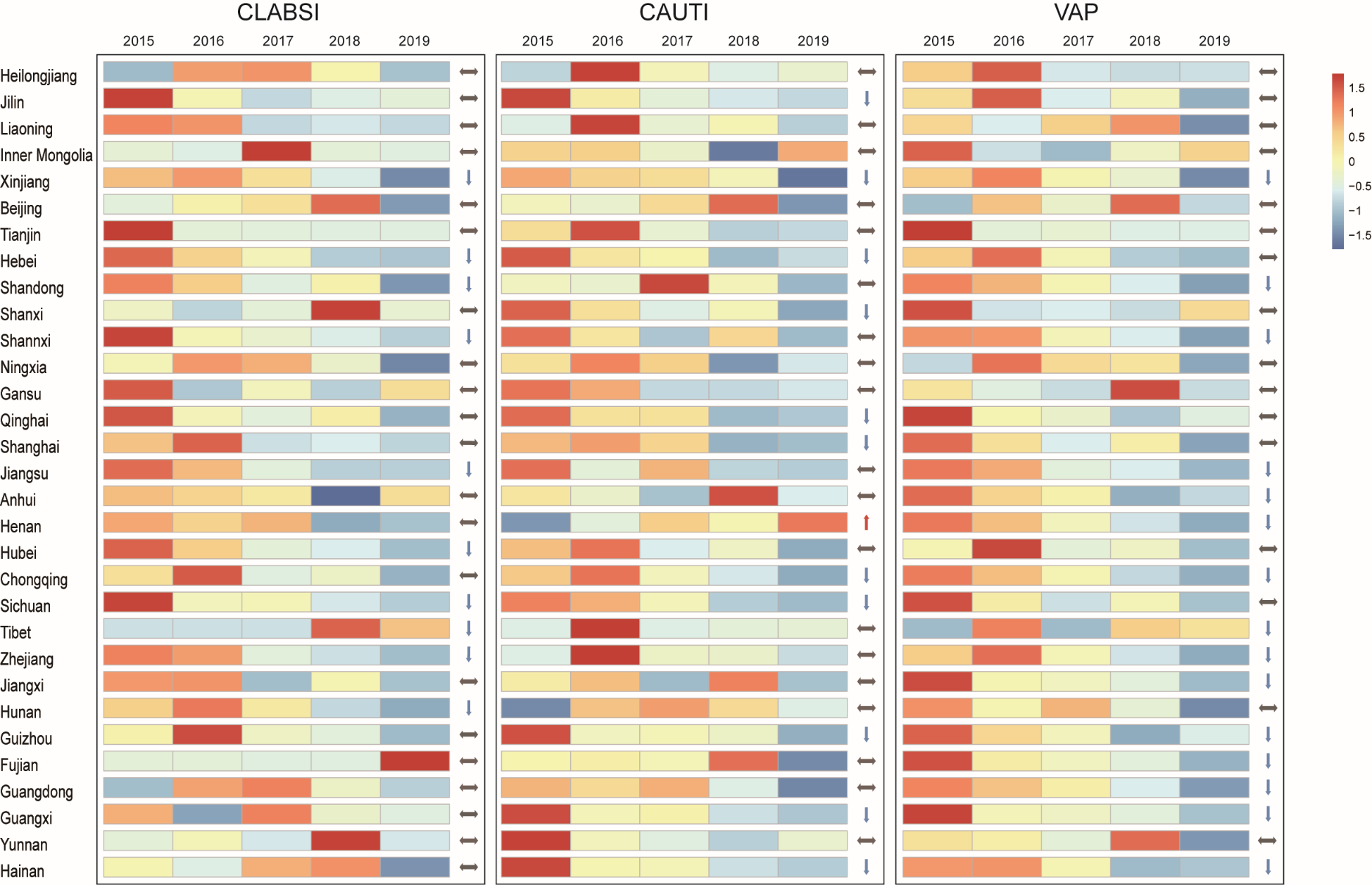
**

**Table S6. Comparison of healthcare-associated infection (HAI) indicators before and after the implementation of the new standards for HAI.**

| **Indicator** | **Median**  **(IQR, %)** | **Multivariate GEE analysis^*^** | |
| --- | --- | --- | --- |
|  |  | **Adjusted IRR (95%CI)** | **p value** |
| Incidence rate of HAIs (%) |  |  |  |
| Before the implementation of the standards | 0.74 (0.37, 1.26) | 1 | - |
| After the implementation of the standards | 0.65 (0.31, 1.14) | 0.88 (0.85, 0.90) | <0.001 |
| Detection rate of CRAB (%) |  |  |  |
| Before the implementation of the standards | 35.7 (13.9, 61.4) | 1 | - |
| After the implementation of the standards | 36.2 (14.3, 59.9) | 1.20 (1.01, 1.42) | 0.035 |
| Detection rate of CREC (%) |  |  |  |
| Before the implementation of the standards | 2.2 (0.9, 9.4) | 1 | - |
| After the implementation of the standards | 2.2 (0.9, 6.1) | 0.89 (0.81, 0.98) | 0.014 |
| Detection rate of CRKP (%) |  |  |  |
| Before the implementation of the standards | 3.6 (0.2, 13.3) | 1 | - |
| After the implementation of the standards | 4.2 (0.6, 14.3) | 1.27 (1.05, 1.53) | 0.015 |
| Detection rate of CRPA (%) |  |  |  |
| Before the implementation of the standards | 10.9 (2.7, 24.1) | 1 | - |
| After the implementation of the standards | 11.2 (3.4, 23.6) | 1.29 (1.08, 1.55) | 0.006 |
| Detection rate of MRSA (%) |  |  |  |
| Before the implementation of the standards | 26.4 (14.8, 40.0) | No significant and excluded | |
| After the implementation of the standards | 25.9 (15.2, 38.0) |  |  |
| Detection rate of VREfs (%) |  |  |  |
| Before the implementation of the standards | 0.0 (0.0, 0.0) | 1 | - |
| After the implementation of the standards | 0.0 (0.0, 0.0) | 0.70 (0.54, 0.90) | 0.005 |
| Detection rate of VREfm (%) |  |  |  |
| Before the implementation of the standards | 0.0 (0.0, 2.3) | 1 | - |
| After the implementation of the standards | 0.0 (0.0, 1.0) | 0.52 (0.39, 0.70) | <0.001 |
| Incidence density of CLABSI (events/1000 days) |  |  |  |
| Before the implementation of the standards | 0.04 (0.00, 0.15) | No significant and excluded | |
| After the implementation of the standards | 0.03 (0.00, 0.11) |  |  |
| Incidence density of CAUTI (events/1000 days) |  |  |  |
| Before the implementation of the standards | 0.13 (0.04, 0.29) | 1 | - |
| After the implementation of the standards | 0.11 (0.02, 0.23) | 0.86 (0.76, 0.97) | 0.017 |
| Incidence density of VAP (events/1000 days) |  |  |  |
| Before the implementation of the standards | 0.7 (0.3, 1.5) | 1 | - |
| After the implementation of the standards | 0.5 (0.1, 1.0) | 0.61 (0.53, 0.69) | <0.001 |

Before the implementation of the standards indicates the period 2015-2017. After the implementation of the standards indicates the period 2018-2019.

* The multivariate GEE analysis is a summary of the results of the independent variable i.e. 12 new standards targeted for HAI in the risk factors analysis of the corresponding HAI indicator.

GEE: generalized estimation equation; HAI: healthcare-associated infection; CRAB: carbapenem-resistant *Acinetobacter baumannii*; CREC: carbapenem-resistant *Escherichia coli*; CRKP: carbapenem-resistant *Klebsiella pneumonia*; CRPA: carbapenem-resistant *Pseudomonas aeruginosa*; MRSA: methicillin-resistant *Staphylococcus aureus*; VREfs: vancomycin-resistant *Enterococcus faecalis*; VREfm: vancomycin-resistant *Enterococcus faecium*; CLABSI: central line-associated bloodstream infection; CAUTI: catheter-associated urinary tract infection; VAP: ventilator-associated pneumonia.

**Figure S4.** **Comparison of annual trend of HAI-related quality indicators (QIs) between different subgroups after adjustment for multiple comparison.**


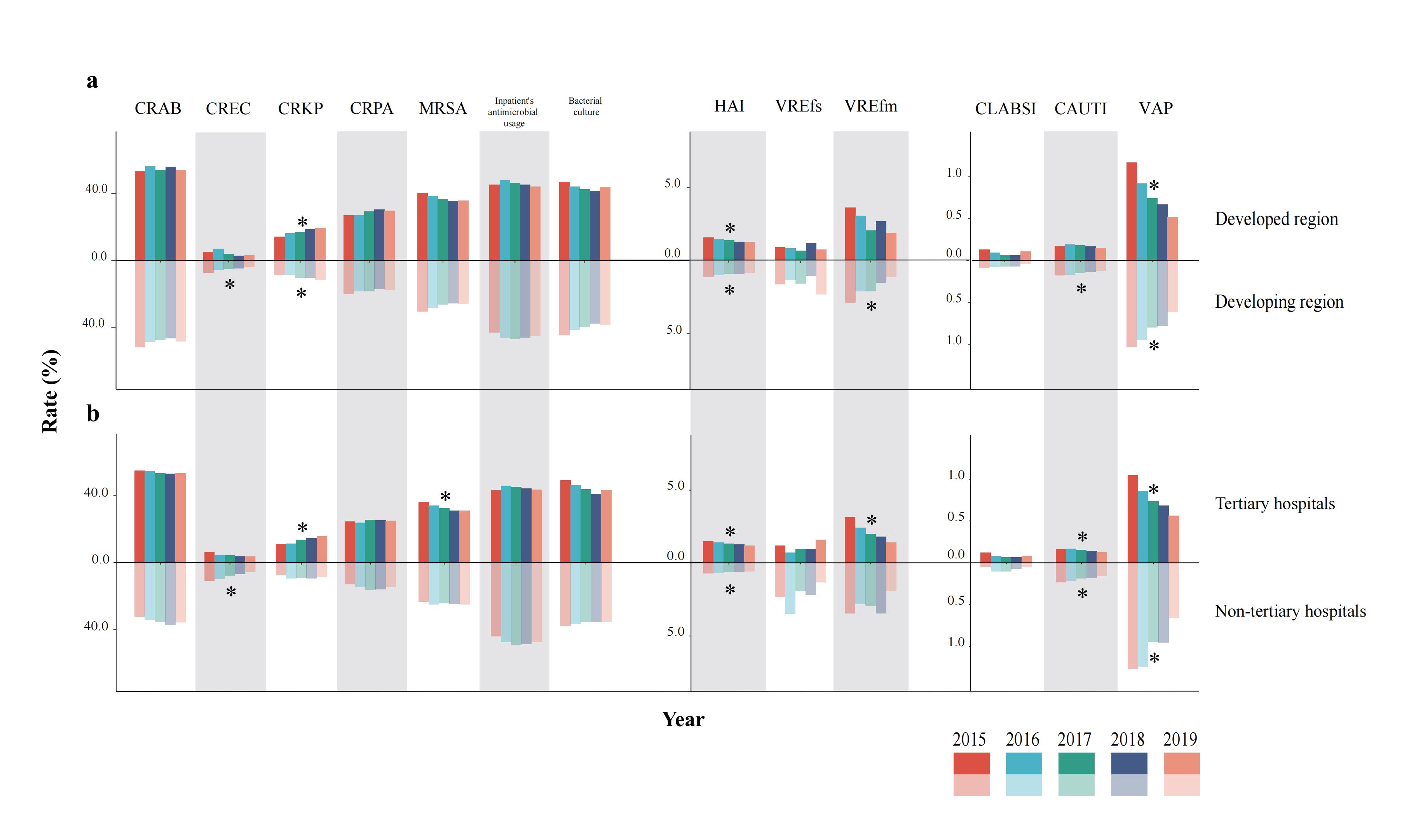


**Table S7. The factors associated with the detection rate of carbapenem-resistant *Acinetobacter baumannii* (CRAB) in China, 2015-2019.**

|  | **No. of hospitals** | **Detection rate,**  **Median (IQR, %)** | **Univariate analysis** | |  | **Multivariate analysis** | |
| --- | --- | --- | --- | --- | --- | --- | --- |
|  |  |  | **Crude IRR (95%CI)** | **p value** |  | **Adjusted IRR (95%CI)** | **p value** |
| Hospital level |  | | | | | | |
| Non-tertiary | 1992 | 23.3 (7.7, 49.0) | 1 | - |  |  | |
| Tertiary | 2048 | 44.9 (23.0, 65.9) | 5.57 (4.36, 7.13) | <0.001 |  |  |  |
| Number of beds |  | | | | | | |
| ＜500 | 1966 | 23.1 (7.1, 48.5) | 1 | - |  | 1 | - |
| 500-1500 | 1757 | 42.4 (21.2, 63.3) | 6.49 (5.02, 8.40) | <0.001 |  | 3.44 (2.63, 4.51) | <0.001 |
| ≥1500 | 240 | 62.5 (41.6, 75.9) | 16.96 (12.35, 23.28) | <0.001 |  | 2.48 (1.62, 3.78) | <0.001 |
| Region of hospital |  | | | | | | |
| North | 1584 | 34.4 (13.3, 60.0) | 1 | - |  | No significant and excluded | |
| South | 2379 | 37.0 (14.3, 60.9) | 1.10 (0.86, 1.42) | 0.454 |  |  |  |
| Urban area where hospital was distributed | | | | | | | |
| No | 1462 | 23.7 (8.0, 49.6) | 1 | - |  | No significant and excluded | |
| Yes | 2355 | 42.3 (19.8, 64.4) | 4.25 (3.24, 5.58) | <0.001 |  |  |  |
| 12 new standards targeted for HAI | | | | | | | |
| Before (2015-2017) | 2949 | 35.7 (13.8, 61.4) | 1 | - |  | 1 | - |
| After (2018-2019) | 3301 | 36.2 (14.3, 59.9) | 1.29 (1.09, 1.52) | 0.003 |  | 1.20 (1.01, 1.42) | 0.035 |
| Annual number of inpatient (10 thousand people), Continuous | | | 1.62 (1.50, 1.75) | <0.001 |  | 1.46 (1.35, 1.57) | <0.001 |
| Annual number of inpatient^2^ | | | 0.992 (0.987, 0.996) | <0.001 |  | 0.993 (0.990, 0.997) | <0.001 |
| <2 | 1581 | 26.8 (7.7, 53.8) |  |  |  |  |  |
| 2−2.99 | 1028 | 30.0 (11.1, 54.4) |  |  |  |  |  |
| 3−3.99 | 842 | 37.0 (16.6, 59.3) |  |  |  |  |  |
| ≥4 | 1147 | 49.4 (28.2, 68.4) |  |  |  |  |  |
| Provincial GDP per capita (10 thousand yuan), Continuous | | | 1.31 (1.17, 1.46) | <0.001 |  | 1.24 (1.11, 1.38) | <0.001 |
| Provincial GDP per capita^2^ | | | 0.990 (0.985, 0.996) | <0.001 |  | 0.992 (0.986, 0.997) | 0.001 |
| <4 | 2117 | 37.0 (14.3, 61.8) |  |  |  |  |  |
| 4−5.99 | 1572 | 32.3 (12.2, 57.0) |  |  |  |  |  |
| 6−7.99 | 986 | 40.8 (18.2, 63.5) |  |  |  |  |  |
| ≥8 | 692 | 33.3 (12.5, 58.1) |  |  |  |  |  |

The positive detection rate of CRAB at the hospital level was used as the outcome variable and hospital level, number of beds, region of hospital, urban area where hospital was distributed, 12 new standards targeted for HAI, annual number of inpatients and provincial GDP per capita were used as independent variables. Univariate and multivariate GEE models were applied.

GDP: gross domestic product; IRR: incidence rate ratio. Hospital level was excluded due to its high correlation with the area (urban/rural) where hospital was distributed.

**Table S8. The factors associated with the detection rate of carbapenem-resistant *Escherichia coli* (CREC) in China, 2015-2019.**

|  | **No. of hospitals** | **Detection rate,**  **Median (IQR, %)** | **Univariate analysis** | |  | **Multivariate analysis** | |
| --- | --- | --- | --- | --- | --- | --- | --- |
|  |  |  | **Crude IRR (95%CI)** | **p value** |  | **Adjusted IRR (95%CI)** | **p value** |
| Hospital level |  | | | | | | |
| Non-tertiary | 2262 | 3.7 (1.2, 14.5) | 1 | - |  |  | |
| Tertiary | 1972 | 1.8 (0.8, 4.2) | 0.71 (0.63, 0.81) | <0.001 |  |  |  |
| Number of beds |  | | | | | | |
| ＜500 | 2241 | 3.3 (1.1, 13.9) | 1 | - |  | No significant and excluded | |
| 500-1500 | 1689 | 1.8 (0.8, 4.7) | 0.80 (0.70, 0.92) | 0.001 |  |  |  |
| ≥1500 | 230 | 1.8 (1.0, 3.5) | 0.80 (0.68, 0.94) | 0.008 |  |  |  |
| Region of hospital |  | | | | | | |
| North | 1656 | 2.6 (1.0, 8.9) | 1 | - |  | 1 | - |
| South | 2504 | 2.0 (0.8, 6.5) | 0.88 (0.77, 1.01) | 0.059 |  | 0.88 (0.77, 1.00) | 0.043 |
| Urban area where hospital was distributed | | | | | | | |
| No | 1640 | 3.6 (1.2, 14.7) | 1 | - |  | 1 | - |
| Yes | 2355 | 1.9 (0.8, 4.8) | 0.71 (0.62, 0.81) | <0.001 |  | 0.70 (0.61, 0.81) | <0.001 |
| 12 new standards targeted for HAI | | | | | | | |
| Before (2015-2017) | 2910 | 2.2 (0.9, 9.4) | 1 | - |  | 1 | - |
| After (2018-2019) | 3326 | 2.2 (0.9, 6.1) | 0.89 (0.81, 0.98) | 0.013 |  | 0.89 (0.81, 0.98) | 0.014 |
| Annual number of inpatient (10 thousand people), Continuous | | | 0.96 (0.93, 0.98) | 0.001 |  | No significant and excluded | |
| Annual number of inpatient^2^ | | | 1.00107 (1.00029, 1.00186) | 0.007 |  |  |  |
| <2 | 1757 | 3.5 (1.3, 12.5) |  |  |  |  |  |
| 2−2.99 | 964 | 2.3 (0.9, 7.9) |  |  |  |  |  |
| 3−3.99 | 760 | 1.8 (0.7, 5.5) |  |  |  |  |  |
| ≥4 | 1094 | 1.5 (0.7, 3.2) |  |  |  |  |  |
| Provincial GDP per capita (10 thousand yuan), Continuous | | | 0.99 (0.97, 1.00) | 0.058 |  | No significant and excluded | |
| <4 | 2226 | 2.4 (0.9, 7.6) |  |  |  |  |  |
| 4−5.99 | 1586 | 2.3 (0.9, 8.5) |  |  |  |  |  |
| 6−7.99 | 972 | 1.9 (0.9, 6.3) |  |  |  |  |  |
| ≥8 | 636 | 1.8 (0.8, 5.4) |  |  |  |  |  |

The positive detection rate of CREC at the hospital level was used as the outcome variable and hospital level, number of beds, region of hospital, urban area where hospital was distributed, 12 new standards targeted for HAI, annual number of inpatients and provincial GDP per capita were used as independent variables. Univariate and multivariate GEE models were applied. The square order of provincial GDP per capita was initially excluded as it failed to meet the inclusion criteria for multivariate analysis (p > 0.2 in univariate analysis).

GDP: gross domestic product; IRR: incidence rate ratio. Hospital level was excluded due to its high correlation with the area (urban/rural) where hospital was distributed.

**Table S9. The factors associated with the detection rate of carbapenem-resistant *Klebsiella pneumonia* (CRKP) in China, 2015-2019.**

|  | **No. of**  **hospitals** | **Detection rate,  Median (IQR, %)** | **Univariate analysis** | |  | **Multivariate analysis** | |
| --- | --- | --- | --- | --- | --- | --- | --- |
|  |  |  | **Crude IRR (95%CI)** | **p value** |  | **Adjusted IRR (95%CI)** | **p value** |
| Hospital level |  | | | | | | |
| Non-tertiary | 2665 | 3.0 (0.0, 11.6) | 1 | - |  |  | |
| Tertiary | 2195 | 4.8 (1.1, 15.7) | 8.19 (6.19, 10.84) | <0.001 |  |  |  |
| Number of beds |  | | | | | | |
| ＜500 | 2655 | 2.8 (0.0, 10.9) | 1 | - |  | 1 | - |
| 500-1500 | 1868 | 4.0 (0.6, 13.7) | 8.40 (6.27, 11.25) | <0.001 |  | 2.74 (1.99, 3.77) | <0.001 |
| ≥1500 | 242 | 9.5 (2.4, 24.1) | 41.51 (27.19, 63.36) | <0.001 |  | 1.60 (0.94, 2.72) | 0.081 |
| Region of hospital |  | | | | | | |
| North | 1939 | 3.6 (0.2, 14.0) | 1 | - |  | No significant and excluded | |
| South | 2826 | 4.0 (0.6, 13.7) | 1.27 (0.94, 1.72) | 0.114 |  |  |  |
| Urban area where hospital was distributed |  | | | | | | |
| No | 1900 | 3.1 (0.0, 11.4) | 1 | - |  | No significant and excluded | |
| Yes | 2631 | 4.5 (0.9, 15.4) | 5.47 (4.04, 7.41) | <0.001 |  |  |  |
| 12 new standards targeted for HAI | | | | | | | |
| Before (2015-2017) | 3569 | 3.6 (0.2, 13.3) | 1 | - |  | 1 | - |
| After (2018-2019) | 3976 | 4.2 (0.6, 14.3) | 1.47 (1.21, 1.78) | <0.001 |  | 1.27 (1.05, 1.53) | 0.015 |
| Annual number of inpatient (10 thousand people), Continuous | | | 1.96 (1.78, 2.16) | <0.001 |  | 1.79 (1.62, 1.97) | <0.001 |
| Annual number of inpatient^2^ | | | 0.99 (0.98, 1.00) | <0.001 |  | 0.99 (0.99, 1.00) | <0.001 |
| <2 | 2163 | 3.3 (0.0, 13.0) |  |  |  |  |  |
| 2−2.99 | 1165 | 3.3 (0.3, 11.8) |  |  |  |  |  |
| 3−3.99 | 889 | 4.0 (0.9, 13.9) |  |  |  |  |  |
| ≥4 | 1162 | 5.0 (1.5, 15.7) |  |  |  |  |  |
| Provincial GDP per capita (10 thousand yuan), Continuous | | | 1.54 (1.36, 1.73) | <0.001 |  | 1.44 (1.29, 1.62) | <0.001 |
| Provincial GDP per capita^2^ | | | 0.99 (0.98, 0.99) | <0.001 |  | 0.99 (0.98, 0.99) | <0.001 |
| <4 | 2576 | 4.6 (0.6, 16.0) |  |  |  |  |  |
| 4−5.99 | 1914 | 3.2 (0.0, 11.3) |  |  |  |  |  |
| 6−7.99 | 1175 | 3.9 (0.7, 12.8) |  |  |  |  |  |
| ≥8 | 820 | 3.5 (0.0, 11.2) |  |  |  |  |  |

The positive detection rate of CRKP at the hospital level was used as the outcome variable and hospital level, number of beds, region of hospital, urban area where hospital was distributed, 12 new standards targeted for HAI, annual number of inpatients and provincial GDP per capita were used as independent variables. Univariate and multivariate GEE models were applied.

GDP: gross domestic product; IRR: incidence rate ratio. Hospital level was excluded due to its high correlation with the area (urban/rural) where hospital was distributed.

**Table S10. The factors associated with the detection rate of carbapenem-resistant *Pseudomonas aeruginosa* (CRPA) in China, 2015-2019.**

|  | **No. of**  **hospitals** | **Detection rate,  Median (IQR, %)** | **Univariate analysis** | |  | **Multivariate analysis** | |
| --- | --- | --- | --- | --- | --- | --- | --- |
|  |  |  | **Crude IRR (95%CI)** | **p value** |  | **Adjusted IRR (95%CI)** | **p value** |
| Hospital level |  | | | | | | |
| Non-tertiary | 2443 | 7.0 (1.0, 18.2) | 1 | - |  |  | |
| Tertiary | 2107 | 14.8 (5.8, 27.7) | 10.06 (7.84, 12.93) | <0.001 |  |  |  |
| Number of beds |  | | | | | | |
| ＜500 | 2401 | 6.9 (0.9, 18.3) | 1 | - |  | 1 | - |
| 500-1500 | 1813 | 13.7 (5.3, 25.9) | 10.85 (8.34, 14.11) | <0.001 |  | 3.82 (2.74, 5.32) | <0.001 |
| ≥1500 | 243 | 23.2 (12.2, 34.7) | 33.50 (23.65, 47.45) | <0.001 |  | 2.62 (1.60, 4.27) | <0.001 |
| Region of hospital |  | | | | | | |
| North | 1787 | 10.8 (2.5, 23.5) | 1 | - |  | 1 | - |
| South | 2670 | 11.2 (3.5, 24.1) | 1.67 (1.27, 2.19) | <0.001 |  | 1.51 (1.17, 1.95) | 0.002 |
| Urban area where hospital was distributed |  | | | | | | |
| No | 1781 | 6.9 (1.1, 17.8) | 1 | - |  | 1 | - |
| Yes | 2488 | 13.9 (4.8, 27.0) | 7.59 (5.74, 10.05) | <0.001 |  | 1.59 (1.13, 2.24) | 0.008 |
| 12 new standards targeted for HAI | | | | | | | |
| Before (2015-2017) | 3315 | 10.9 (2.7, 24.1) | 1 | - |  | 1 | - |
| After (2018-2019) | 3733 | 11.2 (3.4, 23.6) | 1.48 (1.24, 1.78) | <0.001 |  | 1.29 (1.08, 1.55) | 0.006 |
| Annual number of inpatient (10 thousand people), Continuous | | | 1.79 (1.63, 1.95) | <0.001 |  | 1.52 (1.40, 1.65) | <0.001 |
| Annual number of inpatient^2^ | | | 0.99 (0.98, 1.00) | <0.001 |  | 0.99 (0.99, 1.00) | <0.001 |
| <2 | 1942 | 9.8 (1.5, 23.6) |  |  |  |  |  |
| 2−2.99 | 1127 | 8.6 (2.1, 20.5) |  |  |  |  |  |
| 3−3.99 | 879 | 11.3 (3.5, 22.4) |  |  |  |  |  |
| ≥4 | 1170 | 14.9 (6.7, 27.7) |  |  |  |  |  |
| Provincial GDP per capita (10 thousand yuan), Continuous | | | 1.47 (1.33, 1.63) | <0.001 |  | 1.37 (1.24, 1.51) | <0.001 |
| Provincial GDP per capita^2^ | | | 0.99 (0.98, 0.99) | <0.001 |  | 0.99 (0.99, 1.00) | <0.001 |
| <4 | 2431 | 14.0 (3.7, 29.4) |  |  |  |  |  |
| 4−5.99 | 1765 | 9.1 (2.4, 20.9) |  |  |  |  |  |
| 6−7.99 | 1114 | 10.5 (3.9, 21.6) |  |  |  |  |  |
| ≥8 | 776 | 13.6 (5.0, 26.1) |  |  |  |  |  |

The positive detection rate of CRPA at the hospital level was used as the outcome variable and hospital level, number of beds, region of hospital, urban area where hospital was distributed, 12 new standards targeted for HAI, annual number of inpatients and provincial GDP per capita were used as independent variables. Univariate and multivariate GEE models were applied.

GDP: gross domestic product; IRR: incidence rate ratio. Hospital level was excluded due to its high correlation with the area (urban/rural) where hospital was distributed.

**Table S11. The factors associated with the detection rate of methicillin-resistant *Staphylococcus aureus* (MRSA) in China, 2015-2019.**

|  | **No. of hospitals** | **Detection rate,**  **Median (IQR, %)** | **Univariate analysis** | |  | **Multivariate analysis** | |
| --- | --- | --- | --- | --- | --- | --- | --- |
|  |  |  | **Crude IRR (95%CI)** | **p value** |  | **Adjusted IRR (95%CI)** | **p value** |
| Hospital level |  | | | | | | |
| Non-tertiary | 2657 | 22.9 (10.9, 37.0) | 1 | - |  |  | |
| Tertiary | 2215 | 28.6 (18.5, 40.0) | 2.45 (2.13, 2.82) | <0.001 |  |  |  |
| Number of beds |  | | | | | | |
| ＜500 | 2659 | 22.5 (10.9, 36.4) | 1 | - |  | 1 | - |
| 500-1500 | 1872 | 28.3 (18.1, 40.1) | 2.27 (1.96, 2.63) | <0.001 |  | 1.41 (1.17, 1.69) | <0.001 |
| ≥1500 | 242 | 33.1 (23.4, 43.7) | 3.55 (3.02, 4.18) | <0.001 |  | 1.18 (0.92, 1.50) | 0.192 |
| Region of hospital |  | | | | | | |
| North | 1897 | 23.1 (12.9, 36.7) | 1 | - |  | 1 | - |
| South | 2876 | 28.0 (16.7, 40.3) | 1.41 (1.21, 1.65) | <0.001 |  | 1.40 (1.20, 1.63) | <0.001 |
| Urban area where hospital was distributed | | | | | | | |
| No | 1924 | 22.9 (10.9, 36.0) | 1 | - |  | 1 | - |
| Yes | 2621 | 28.1 (17.7, 40.3) | 2.21 (1.88, 2.59) | <0.001 |  | 1.36 (1.11, 1.67) | 0.003 |
| 12 new standards targeted for HAI | | | | | | | |
| Before (2015-2017) | 3573 | 26.4 (14.8, 40.0) | 1 | - |  | No significant and excluded | |
| After (2018-2019) | 3981 | 25.9 (15.2, 38.0) | 1.15 (1.04, 1.28) | 0.009 |  |  |  |
| Annual number of inpatient (10 thousand people), Continuous | | | 1.25 (1.20, 1.31) | <0.001 |  | 1.19 (1.14, 1.25) | <0.001 |
| Annual number of inpatient^2^ | | | 1.00 (0.99, 1.00) | 0.002 |  | 1.00 (0.99, 1.00) | 0.006 |
| <2 | 2160 | 24.1 (12.0, 39.1) |  |  |  |  |  |
| 2−2.99 | 1205 | 24.3 (13.3, 37.0) |  |  |  |  |  |
| 3−3.99 | 901 | 26.3 (16.4, 38.2) |  |  |  |  |  |
| ≥4 | 1179 | 29.5 (20.2, 40.0) |  |  |  |  |  |
| Provincial GDP per capita (10 thousand yuan), Continuous | | | 1.15 (1.09, 1.22) | <0.001 |  | 1.07 (1.02, 1.13) | 0.011 |
| Provincial GDP per capita^2^ | | | 0.997 (0.995, 0.999) | 0.033 |  | 0.99815 (0.99585, 1.00046) | 0.117 |
| <4 | 2594 | 28.1 (16.4, 41.3) |  |  |  |  |  |
| 4−5.99 | 1906 | 24.3 (13.0, 37.4) |  |  |  |  |  |
| 6−7.99 | 1181 | 25.0 (14.8, 37.0) |  |  |  |  |  |
| ≥8 | 828 | 24.5 (15.4, 35.1) |  |  |  |  |  |

The positive detection rate of MRSA at the hospital level was used as the outcome variable and hospital level, number of beds, region of hospital, urban area where hospital was distributed, 12 new standards targeted for HAI, annual number of inpatients and provincial GDP per capita were used as independent variables. Univariate and multivariate GEE models were applied.

GDP: gross domestic product; IRR: incidence rate ratio. Hospital level was excluded due to its high correlation with the area (urban/rural) where hospital was distributed.

**Table S12. The factors associated with the detection rate of vancomycin-resistant *Enterococcus faecalis* (VREfs) in China, 2015-2019.**

|  | **No. of**  **hospitals** | **Detection rate,  Median (IQR, %)** | **Univariate analysis** | |  | **Multivariate analysis** | |
| --- | --- | --- | --- | --- | --- | --- | --- |
|  |  |  | **Crude IRR (95%CI)** | **p value** |  | **Adjusted IRR (95%CI)** | **p value** |
| Hospital level |  | | | | | | |
| Non-tertiary | 1227 | 0.0 (0.0, 0.0) | 1 | - |  |  | |
| Tertiary | 1747 | 0.0 (0.0, 0.0) | 0.88 (0.63, 1.21) | 0.424 |  |  |  |
| Number of beds |  | | | | | | |
| ＜500 | 1230 | 0.0 (0.0, 0.0) | 1 | - |  | No significant and excluded | |
| 500-1500 | 1463 | 0.0 (0.0, 0.0) | 0.89 (0.64, 1.24) | 0.488 |  |  |  |
| ≥1500 | 227 | 0.0 (0.0, 0.6) | 2.26 (1.25, 4.06) | 0.007 |  |  |  |
| Region of hospital |  | | | | | | |
| North | 1043 | 0.0 (0.0, 0.0) | 1 | - |  | No significant and excluded | |
| South | 1877 | 0.0 (0.0, 0.0) | 0.86 (0.62, 1.20) | 0.373 |  |  |  |
| Urban area where hospital was distributed | | | | | | | |
| No | 883 | 0.0 (0.0, 0.0) | 1 | - |  | No significant and excluded | |
| Yes | 1956 | 0.0 (0.0, 0.0) | 0.91 (0.63, 1.30) | 0.597 |  |  |  |
| 12 new standards targeted for HAI | | | | | | | |
| Before (2015-2017) | 2057 | 0.0 (0.0, 0.0) | 1 | - |  | 1 | - |
| After (2018-2019) | 2398 | 0.0 (0.0, 0.0) | 0.72 (0.56, 0.92) | 0.010 |  | 0.70 (0.54, 0.90) | 0.005 |
| Annual number of inpatient (10 thousand people), Continuous | | | 1.09 (1.05, 1.14) | <0.001 |  | 1.10 (1.05, 1.15) | <0.001 |
| <2 | 940 | 0.0 (0.0, 0.0) |  |  |  |  |  |
| 2−2.99 | 728 | 0.0 (0.0, 0.0) |  |  |  |  |  |
| 3−3.99 | 660 | 0.0 (0.0, 0.0) |  |  |  |  |  |
| ≥4 | 1034 | 0.0 (0.0, 0.0) |  |  |  |  |  |
| Provincial GDP per capita (10 thousand yuan), Continuous | | | 1.01 (0.98, 1.05) | 0.555 |  | No significant and excluded | |
| <4 | 1571 | 0.0 (0.0, 0.0) |  |  |  |  |  |
| 4−5.99 | 1033 | 0.0 (0.0, 0.0) |  |  |  |  |  |
| 6−7.99 | 740 | 0.0 (0.0, 0.0) |  |  |  |  |  |
| ≥8 | 510 | 0.0 (0.0, 0.0) |  |  |  |  |  |

The positive detection rate of VREfs at the hospital level was used as the outcome variable and hospital level, number of beds, region of hospital, urban area where hospital was distributed, 12 new standards targeted for HAI, annual number of inpatients and provincial GDP per capita were used as independent variables. Univariate and multivariate GEE models were applied. The square orders of annual number of inpatient and provincial GDP per capita were initially excluded as they failed to meet the inclusion criteria for multivariate analysis (both p > 0.2 in univariate analysis).

GDP: gross domestic product; IRR: incidence rate ratio. Hospital level was excluded due to its high correlation with the area (urban/rural) where hospital was distributed.

**Table S13. The factors associated with the detection rate of vancomycin-resistant *Enterococcus faecium* (VREfm) in China, 2015-2019.**

|  | **No. of**  **hospitals** | **Detection rate,  Median (IQR, %)** | **Univariate analysis** | |  | **Multivariate analysis** | |
| --- | --- | --- | --- | --- | --- | --- | --- |
|  |  |  | **Crude IRR (95%CI)** | **p value** |  | **Adjusted IRR (95%CI)** | **p value** |
| Hospital level |  | | | | | | |
| Non-tertiary | 1086 | 0.0 (0.0, 1.2) | 1 | - |  |  | |
| Tertiary | 1734 | 0.0 (0.0, 1.6) | 2.65 (1.78, 3.95) | <0.001 |  |  |  |
| Number of beds |  | | | | | | |
| ＜500 | 1089 | 0.0 (0.0, 0.0) | 1 | - |  | 1 | - |
| 500-1500 | 1457 | 0.0 (0.0, 0.0) | 2.32 (1.55, 3.47) | <0.001 |  | 1.34 (0.89, 2.04) | 0.165 |
| ≥1500 | 227 | 0.4 (0.0, 2.1) | 21.37 (10.48, 43.58) | <0.001 |  | 4.36 (1.90, 10.03) | <0.001 |
| Region of hospital |  | | | | | | |
| North | 1066 | 0.0 (0.0, 2.2) | 1 | - |  | 1 | - |
| South | 1707 | 0.0 (0.0, 1.3) | 0.62 (0.41, 0.93) | 0.020 |  | 0.55 (0.37, 0.83) | 0.004 |
| Urban area where hospital was distributed |  | | | | | | |
| No | 794 | 0.0 (0.0, 1.2) | 1 | - |  | No significant and excluded | |
| Yes | 1910 | 0.0 (0.0, 1.6) | 2.31 (1.51, 3.55) | <0.001 |  |  |  |
| 12 new standards targeted for HAI | | | | | | | |
| Before (2015-2017) | 1941 | 0.0 (0.0, 2.3) | 1 | - |  | 1 | - |
| After (2018-2019) | 2273 | 0.0 (0.0, 1.0) | 0.62 (0.47, 0.83) | 0.001 |  | 0.52 (0.39, 0.70) | <0.001 |
| Annual number of inpatient (10 thousand people), Continuous | | | 1.33 (1.23, 1.43) | <0.001 |  | 1.23 (1.13, 1.34) | <0.001 |
| Annual number of inpatient^2^ | | | 0.998 (0.996, 0.999) | 0.005 |  | 0.99838 (0.99676, 1.00001) | 0.052 |
| <2 | 846 | 0.0 (0.0, 1.7) |  |  |  |  |  |
| 2−2.99 | 693 | 0.0 (0.0, 2.1) |  |  |  |  |  |
| 3−3.99 | 646 | 0.0 (0.0, 1.5) |  |  |  |  |  |
| ≥4 | 1023 | 0.0 (0.0, 1.5) |  |  |  |  |  |
| Provincial GDP per capita (10 thousand yuan), Continuous | | | 1.11 (1.06, 1.16) | <0.001 |  | 1.30 (1.12, 1.51) | <0.001 |
| Provincial GDP per capita^2^ | | | 0.99 (0.98, 1.00) | 0.011 |  | 0.99 (0.99, 1.00) | 0.071 |
| <4 | 1506 | 0.0 (0.0, 2.1) |  |  |  |  |  |
| 4−5.99 | 991 | 0.0 (0.0, 1.1) |  |  |  |  |  |
| 6−7.99 | 700 | 0.0 (0.0, 1.3) |  |  |  |  |  |
| ≥8 | 483 | 0.0 (0.0, 1.2) |  |  |  |  |  |

The positive detection rate of VREfs at the hospital level was used as the outcome variable and hospital level, number of beds, region of hospital, urban area where hospital was distributed, 12 new standards targeted for HAI, annual number of inpatients and provincial GDP per capita were used as independent variables. Univariate and multivariate GEE models were applied.

GDP: gross domestic product; IRR: incidence rate ratio. Hospital level was excluded due to its high correlation with the area (urban/rural) where hospital was distributed.

**Table S14. The factors associated with the incidence density of central line-associated bloodstream infection (CLABSI) in China, 2015-2019.**

|  | **No. of hospitals** | **Incidence density,**  **Median (IQR, ‰)** | **Univariate analysis** | |  | **Multivariate analysis** | |
| --- | --- | --- | --- | --- | --- | --- | --- |
|  |  |  | **Crude IRR (95%CI)** | **p value** |  | **Adjusted IRR (95%CI)** | **p value** |
| Hospital level |  | | | | | | |
| Non-tertiary | 2462 | 0.0 (0.0, 0.1) | 1 | - |  |  | |
| Tertiary | 2097 | 0.1 (0.0, 0.1) | 13.36 (10.71, 16.67) | <0.001 |  |  |  |
| Number of beds |  | | | | | | |
| ＜500 | 2443 | 0.0 (0.0, 0.1) | 1 | - |  | 1 | - |
| 500-1500 | 1802 | 0.1 (0.0, 0.1) | 10.59 (8.39, 13.37) | <0.001 |  | 2.88 (2.15, 3.87) | <0.001 |
| ≥1500 | 240 | 0.07 (0.03, 0.15) | 50.21 (34.91, 72.20) | <0.001 |  | 2.07 (1.26, 3.40) | 0.004 |
| Region of hospital |  | | | | | | |
| North | 1826 | 0.01 (0.00, 0.11) | 1 | - |  | 1 | - |
| South | 2642 | 0.04 (0.00, 0.13) | 2.69 (2.11, 3.43) | <0.001 |  | 2.37 (1.90, 2.95) | <0.001 |
| Urban area where hospital was distributed | | | | | | | |
| No | 1868 | 0.0 (0.0, 0.1) | 1 | - |  | 1 | - |
| Yes | 2600 | 0.05 (0.00, 0.13) | 8.46 (6.68, 10.71) | <0.001 |  | 2.09 (1.58, 2.77) | <0.001 |
| 12 new standards targeted for HAI | | | | | | | |
| Before (2015-2017) | 3709 | 0.04 (0.00, 0.15) | 1 | - |  | No significant and excluded | |
| After (2018-2019) | 4088 | 0.02 (0.00, 0.11) | 1.02 (0.87, 1.19) | 0.828 |  |  |  |
| Annual number of inpatient (10 thousand people), Continuous | | | 1.90 (1.75, 2.07) | <0.001 |  | 1.62 (1.50, 1.76) | <0.001 |
| Annual number of inpatient^2^ | | | 0.99 (0.98, 1.00) | <0.001 |  | 0.99 (0.99, 1.00) | <0.001 |
| <2 | 2165 | 0.0 (0.0, 0.1) |  |  |  |  |  |
| 2−2.99 | 1243 | 0.02 (0.00, 0.13) |  |  |  |  |  |
| 3−3.99 | 942 | 0.04 (0.00, 0.14) |  |  |  |  |  |
| ≥4 | 1196 | 0.06 (0.02, 0.14) |  |  |  |  |  |
| Provincial GDP per capita (10 thousand yuan), Continuous | | | 1.42 (1.29, 1.55) | <0.001 |  | 1.27 (1.17, 1.38) | <0.001 |
| Provincial GDP per capita^2^ | | | 0.990 (0.986, 0.994) | <0.001 |  | 0.99 (0.99, 1.00) | <0.001 |
| <4 | 2405 | 0.0 (0.0, 0.1) |  |  |  |  |  |
| 4−5.99 | 1789 | 0.02 (0.00, 0.13) |  |  |  |  |  |
| 6−7.99 | 1113 | 0.04 (0.00, 0.14) |  |  |  |  |  |
| ≥8 | 757 | 0.05 (0.00, 0.13) |  |  |  |  |  |

The incidence density of CLABSI at the hospital level was used as the outcome variable and hospital level, number of beds, region of hospital, urban area where hospital was distributed, 12 new standards targeted for HAI, annual number of inpatients and provincial GDP per capita were used as independent variables. Univariate and multivariate GEE models were applied.

GDP: gross domestic product; IRR: incidence rate ratio. Hospital level was excluded due to its high correlation with the area (urban/rural) where hospital was distributed.

**Table S15. The factors associated with the incidence density of catheter-associated urinary tract infection (CAUTI) in China, 2015-2019.**

|  | **No. of hospitals** | **Incidence density,**  **Median (IQR, ‰)** | **Univariate analysis** | |  | **Multivariate analysis** | |
| --- | --- | --- | --- | --- | --- | --- | --- |
|  |  |  | **Crude IRR (95%CI)** | **p value** |  | **Adjusted IRR (95%CI)** | **p value** |
| Hospital level |  | | | | | | |
| Non-tertiary | 4169 | 0.1 (0.0, 0.3) | 1 | - |  |  | |
| Tertiary | 2350 | 0.1 (0.1, 0.2) | 3.42 (2.84, 4.11) | <0.001 |  |  |  |
| Number of beds |  | | | | | | |
| ＜500 | 3396 | 0.1 (0.0, 0.3) | 1 | - |  | 1 | - |
| 500-1500 | 1884 | 0.1 (0.1, 0.3) | 4.02 (3.32, 4.87) | <0.001 |  | 1.99 (1.59, 2.48) | <0.001 |
| ≥1500 | 241 | 0.1 (0.1, 0.2) | 6.38 (4.86, 8.38) | <0.001 |  | 0.92 (0.63, 1.35) | 0.678 |
| Region of hospital |  | | | | | | |
| North | 2359 | 0.1 (0.0, 0.3) | 1 | - |  | 1 | - |
| South | 3145 | 0.13 (0.04, 0.26) | 2.01 (1.65, 2.45) | <0.001 |  | 1.79 (1.48, 2.18) | <0.001 |
| Urban area where hospital was distributed | | | | | | | |
| No | 2638 | 0.1 (0.0, 0.3) | 1 | - |  | No significant and excluded | |
| Yes | 2866 | 0.12 (0.04, 0.24) | 2.18 (1.79, 2.66) | <0.001 |  |  |  |
| 12 new standards targeted for HAI | | | | | | | |
| Before (2015-2017) | 4750 | 0.13 (0.04, 0.29) | 1 | - |  | 1 | - |
| After (2018-2019) | 5268 | 0.11 (0.02, 0.23) | 0.93 (0.82, 1.05) | 0.235 |  | 0.86 (0.76, 0.97) | 0.017 |
| Annual number of inpatient (10 thousand people), Continuous | | | 1.52 (1.40, 1.65) | <0.001 |  | 1.44 (1.32, 1.56) | <0.001 |
| Annual number of inpatient^2^ | | | 0.99 (0.99, 1.00) | 0.004 |  | 0.99 (0.99, 1.00) | 0.005 |
| <2 | 3161 | 0.1 (0.0, 0.3) |  |  |  |  |  |
| 2−2.99 | 1327 | 0.13 (0.04, 0.28) |  |  |  |  |  |
| 3−3.99 | 961 | 0.1 (0.1, 0.3) |  |  |  |  |  |
| ≥4 | 1201 | 0.1 (0.1, 0.2) |  |  |  |  |  |
| Provincial GDP per capita (10 thousand yuan), Continuous | | | 1.06 (1.04, 1.09) | <0.001 |  | 1.03 (1.01, 1.05) | 0.010 |
| <4 | 2918 | 0.1 (0.0, 0.3) |  |  |  |  |  |
| 4−5.99 | 2241 | 0.11 (0.02, 0.27) |  |  |  |  |  |
| 6−7.99 | 1324 | 0.12 (0.03, 0.27) |  |  |  |  |  |
| ≥8 | 892 | 0.1 (0.1, 0.3) |  |  |  |  |  |

The incidence density of CAUTI at the hospital level was used as the outcome variable and hospital level, number of beds, region of hospital, urban area where hospital was distributed, 12 new standards targeted for HAI, annual number of inpatients and provincial GDP per capita were used as independent variables. Univariate and multivariate GEE models were applied. The square order of provincial GDP per capita was initially excluded as it failed to meet the inclusion criteria for multivariate analysis (p > 0.2 in univariate analysis).

GDP: gross domestic product; IRR: incidence rate ratio. Hospital level was excluded due to its high correlation with the area (urban/rural) where hospital was distributed.

**Table S16. The factors associated with the incidence density of ventilator-associated pneumonia (VAP) in China, 2015-2019.**

|  | **No. of hospitals** | **Incidence density,**  **Median (IQR, ‰)** | **Univariate analysis** | |  | **Multivariate analysis** | |
| --- | --- | --- | --- | --- | --- | --- | --- |
|  |  |  | **Crude IRR (95%CI)** | **p value** |  | **Adjusted IRR (95%CI)** | **p value** |
| Hospital level |  | | | | | | |
| Non-tertiary | 2067 | 0.6 (0.3, 1.1) | 1 | - |  |  | |
| Tertiary | 2192 | 0.6 (0.1, 1.3) | 3.69 (2.95, 4.61) | <0.001 |  |  |  |
| Number of beds |  | | | | | | |
| ＜500 | 2168 | 0.6 (0.1, 1.2) | 1 | - |  | 1 | - |
| 500-1500 | 1780 | 0.7 (0.3, 1.2) | 4.88 (3.87, 6.14) | <0.001 |  | 2.48 (1.94, 3.15) | <0.001 |
| ≥1500 | 237 | 0.6 (0.3, 1.0) | 6.68 (4.71, 9.48) | <0.001 |  | 0.71 (0.45, 1.11) | 0.129 |
| Region of hospital |  | | | | | | |
| North | 1698 | 0.6 (0.2, 1.2) | 1 | - |  | 1 | - |
| South | 2470 | 0.7 (0.3, 1.2) | 2.32 (1.83, 2.96) | <0.001 |  | 2.28 (1.82, 2.87) | <0.001 |
| Urban area where hospital was distributed | | | | | | | |
| No | 1700 | 0.6 (0.2, 1.4) | 1 | - |  | No significant and excluded | |
| Yes | 2468 | 0.6 (0.3, 1.2) | 2.40 (1.88, 3.07) | <0.001 |  |  |  |
| 12 new standards targeted for HAI | | | | | | | |
| Before (2015-2017) | 3480 | 0.7 (0.3, 1.5) | 1 | - |  | 1 | - |
| After (2018-2019) | 3848 | 0.5 (0.1, 1.0) | 0.65 (0.57, 0.75) | <0.001 |  | 0.61 (0.53, 0.69) | <0.001 |
| Annual number of inpatient (10 thousand people), Continuous | | | 1.63 (1.48, 1.79) | <0.001 |  | 1.57 (1.44, 1.72) | <0.001 |
| Annual number of inpatient^2^ | | | 0.991 (0.985, 0.997) | 0.002 |  | 0.992 (0.987, 0.997) | 0.002 |
| <2 | 1845 | 0.5 (0.0, 1.1) |  |  |  |  |  |
| 2−2.99 | 1262 | 0.7 (0.2, 1.3) |  |  |  |  |  |
| 3−3.99 | 944 | 0.7 (0.3, 1.4) |  |  |  |  |  |
| ≥4 | 1206 | 0.7 (0.4, 1.2) |  |  |  |  |  |
| Provincial GDP per capita (10 thousand yuan), Continuous | | | 1.00 (0.999, 1.000) | 0.455 |  | No significant and excluded | |
| <4 | 2226 | 0.6 (0.2, 1.2) |  |  |  |  |  |
| 4−5.99 | 1713 | 0.6 (0.2, 1.3) |  |  |  |  |  |
| 6−7.99 | 1051 | 0.7 (0.3, 1.2) |  |  |  |  |  |
| ≥8 | 711 | 0.5 (0.2, 1.2) |  |  |  |  |  |

The incidence density of VAP at the hospital level was used as the outcome variable and hospital level, number of beds, region of hospital, urban area where hospital was distributed, 12 new standards targeted for HAI, annual number of inpatients and provincial GDP per capita were used as independent variables. Univariate and multivariate GEE models were applied. The square order of provincial GDP per capita was initially excluded as it failed to meet the inclusion criteria for multivariate analysis (p > 0.2 in univariate analysis).

GDP: gross domestic product; IRR: incidence rate ratio. Hospital level was excluded due to its high correlation with the area (urban/rural) where hospital was distributed.

**Figure S5. Trend of current point prevalence survey from 2001 to 2024.** This figure is taken from a report at an annual academic conference on HAIs in China (non-public data, available fromhttps://baijiahao.baidu.com/s?id=1833881590072923240&wfr=spider&for=pc).


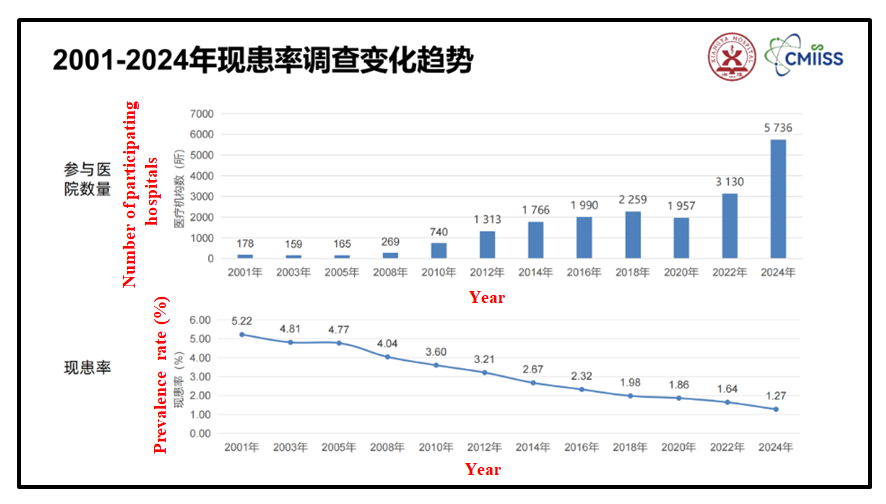

Supplement: Supplemental Data (Clean) [file mmc1.docx]
