# Supplementary material for: Mapping the landscape of healthcare-associated infections in China, 2015–2019: a nation-wide observational study
Source: Lancet Reg Health West Pac. 2025 Dec 16;65:101775. doi: 10.1016/j.lanwpc.2025.101775 (PMC12768928; doi:10.1016/j.lanwpc.2025.101775)
Supplement: Translated Abstract Final [file mmc2.docx]

**Translated Abstract**:

**背景**： 由于抗生素耐药性的不断出现和多重耐药菌不断流行等因素的影响，医院感染（HAIs）的流行及威胁也越来越大，发展中国家尤为突出。本研究旨在分析中国HAIs及其多重耐药菌感染（MDROs）的流行特征、时空异质性和相关因素。

**方法**： 我们纳入了2015−2019年中国6867家医院上报的HAI管理质量控制指标监测数据，通过描述性分析、趋势分析等方法，评估了HAI的年发病率等关键质量指标及其影响因素。

**结果**： 2015-2019年期间，中国6867家医院共报告了4,959,230例HAIs，总体发病率和现患率估计分别为1.1%和2.3%，且呈逐年下降趋势。耐碳青霉烯鲍曼不动杆菌（CRAB）的检出率最高（51.1%），其次为耐甲氧西林金黄色葡萄球菌（MRSA）（31.0%）、耐碳青霉烯铜绿假单胞菌（CRPA）（22.8%）和耐碳青霉烯肺炎克雷伯菌（CRKP）（12.4%）。MRSA、耐碳青霉烯类大肠杆菌（CREC）和耐万古霉素粪肠球菌（VREfm）的检出率明显下降，而CRKP呈显著上升趋势。经济发达地区的医院（城市：发病率比[IRR]=1.39，p<0.001；人均GDP较高地区的医院IRR=1.03，p<0.001）发生HAIs的风险更高；床位数≥500的医院（500−1 500床：IRR=1.64；≥1 500床：IRR=2.16，p均<0.001）及位于南方地区的医院（IRR=1.23，p<0.001）的HAIs发生风险显著升高。2017年国家卫健康委实施12项医院感染防控新标准后，HAIs年发病率显著降低（IRR=0.92，p<0.001）。

**解释**： 本研究为深入理解医院感染及抗生素耐药性的流行现状问题提供了证据支持，明确了HAIs及MDROs的高风险区域，提示需针对性地强化感染管理策略与政策落实。

**基金资助**： 病原微生物生物安全全国重点实验室开放课题基金项目（SKLPBS2443）和“感·动中国”感染防控研究项目（批准号：GY2023022-A）。

**关键词**： 医院感染；多重耐药菌; 危险因素；时空异质性；抗菌药物使用

Disclaimer: This translation in Chinese was submitted by the authors and we reproduce it as supplied. It has not been peer reviewed. Our editorial processes have only been applied to the original abstract in English, which should serve as reference for this manuscript.
